# Dietary *Pelargonium Sidoides* extract mitigates thermal stress in *Oreochromis niloticus*: physiological and immunological insights

**DOI:** 10.1007/s11259-025-10705-z

**Published:** 2025-03-24

**Authors:** Nagwa I. S. Abu-Zahra, Ayman A. Atia, Mohamed M. Elseify, Mona E. Abass, Shireen Soliman

**Affiliations:** 1 Fish Diseases Unit, Kafrelsheikh Provincial Lab, Agriculture Research Center (ARC), Animal Health Research Institute (AHRI), Giza, Egypt; 2 Pathology Unit, Kafrelsheikh Provincial Lab, Agriculture Research Center (ARC), Animal Health Research Institute (AHRI), Giza, Egypt; 3 Immunology Unit, Kafrelsheikh Provincial Lab, Agriculture Research Center (ARC), Animal Health Research Institute (AHRI), Giza, Egypt; 4 Biochemistry, Nutritional Deficiency Diseases and Toxicology Unit, Kafrelsheikh Provincial Lab, Animal Health Research Institute, Agricultural Research Center (ARC), Giza, Egypt

**Keywords:** Aquaculture, Immune response, Nutritional supplements, Phytogenic feed additives, Oxidative stress, Stress mitigation

## Abstract

Extreme water temperatures caused by climate change constitute a serious threat to aquaculture producers. Aquatic ectotherms, which are unable to regulate their body temperature, are highly affected by temperature changes and serve as excellent models for studying the effects of these factors. This study aimed to examine the impact of extreme temperature variation on the growth, biological indices, antioxidant capacity, histology, and disease resistance of *Oreochromis niloticus*. The fish were exposed to 17 °C, 25 °C (control), or 33 °C for 30 days, imitating extreme temperature events. Additionally, a mitigation trial was conducted via dietary *Pelargonium sidoides* root extract (PS). Compared with that at 25 °C, growth performance was significantly impaired by thermal stress. The exposure of *O. niloticus* to thermal stress, especially cold stress, elicited significant increases in stress biomarkers (cortisol and glucose), hepatorenal functions (AST, ALT, LDH, and creatinine), and lipid profiles (triglyceride, cholesterol, HDL, and LDL). Cold stress induced immunosuppression in terms of reduced total protein, phagocytosis, serum bacterial activity, total immunoglobulin, and white blood cells, all of which increased the susceptibility of the fish to *Aeromonas hydrophila* infection and increased mortality. In addition to severe pathological lesions in the gills, liver, and intestine, thermal stress causes an imbalance in antioxidant/oxidative stress. Nevertheless, the PS extract counteracted these detrimental impacts of thermal stress. In conclusion, PS may be recommended for enhancing immunity and antioxidative resistance to mitigate the effects of thermal stress on *O. niloticus*. These findings may be useful in developing hypotheses about the physiology and immunology of fish in extreme-temperature environments.

**Supplementary Information:**

The online version contains supplementary material available at 10.1007/s11259-025-10705-z.

## Introduction

Climate variability and extreme temperature events are increasingly impacting aquaculture systems worldwide. Recent studies have highlighted the significant effects of climate change on aquaculture production, emphasizing the need for adaptive strategies to mitigate these impacts (Maulu et al. 2021). For example, Maulu et al. (2021) reviewed the potential effects of climate change on aquaculture sustainability, noting that rising temperatures pose substantial challenges to the sector. Global temperature patterns have fluctuated significantly due to climate change, and these changes are expected to continue and intensify (Islam et al. 2021). An analysis of temperature trends revealed that the overall area of the world land surface subject to extreme heat and cold is growing (Islam et al. 2021).

*Oreochromis niloticus* is one of the most extensively cultured fishes worldwide, mostly because of its high growth performance, widespread market acceptance, and ability to tolerate a wide variety of temperatures. Recent climate data have revealed notable temperature anomalies in aquaculture-producing regions, with recorded temperatures often exceeding the critical thermal maximum (CTmax) of *Oreochromis niloticus*, which ranges from 43.1 °C to 44.8 °C (Akrokoh et al. 2024). For example, regions such as the Mediterranean, including Spain, Italy, and Turkey, have experienced unprecedented heatwaves, with temperatures occasionally surpassing 45 °C (Reid et al. 2019). Similarly, parts of Egypt and Vietnam have reported temperature fluctuations above the thermal tolerance of *O. niloticus*, posing significant risks to aquaculture operations (Reid et al. 2019). Sudden temperature decreases in Egypt and China have reached 5–8 °C, far exceeding the tolerance limits of *O. niloticus* and leading to high mortality rates (Reid et al. 2019). These anomalies not only challenge the physiological limits of *O. niloticus* but also highlight the urgent need for adaptive strategies to mitigate thermal stress and ensure the sustainability of aquaculture systems.

Hyperthermia impacts organisms at many levels, including physiological, biochemical, molecular, and behavioral levels (Kazmi et al. 2022). Like mammals, fish are protected from immunological damage and inflammatory responses via their innate immunity, which acts as the initial line of defense against hyperthermia stress (Alfons et al. 2023). High temperatures pose a significant threat to the sustainability of aquaculture, particularly affecting the growth, health, and survival of fish species (Khieokhajonkhet et al. 2022). The authors showed that exposure to high temperatures can lead to a range of physiological and biochemical stress responses in fish, including increased metabolic rates and changes in blood biochemistry. Elevated temperatures can also disrupt critical biological processes such as reproduction, feeding, and respiration, leading to reduced productivity in aquaculture systems (Khieokhajonkhet et al. 2022). Moreover, high temperatures can exacerbate the proliferation of pathogens and parasites, further compromising fish health and increasing disease susceptibility (Jeffries et al. 2016). Understanding these impacts is crucial for developing adaptive management practices to mitigate the adverse effects of high-temperature stress on fish, ensuring the sustainability and productivity of aquaculture under changing climatic conditions (Yadav et al. 2024).

*O. niloticus* generally starts to experience cold stress at temperatures below 18 °C (Behairy et al. 2023). According to Wu et al. (2019), it cannot tolerate temperatures below 10 °C for longer than a few days and cannot grow well below 18 °C. *O. niloticus* susceptibility to cold temperatures is a serious economic concern because it limits their growth seasons and results in significant overwinter mortality (Liu et al. 2022). Hypothermia may impair fish immunological responses, change blood enzyme levels, increase cortisol levels, and slow metabolism (Behairy et al. 2023). Fish that are stressed by cold water migrate deeper into ponds, congregate in large numbers, stop feeding, become lethargic, and are more vulnerable to predators (Behairy et al. 2023). According to Zhang et al. (2023), low water temperatures can also have an adverse effect on nutritional digestibility by slowing digestion, prolonging the time in the gut, and reducing the rate of gastrointestinal emptying. Furthermore, in response to pathogens, hypothermia inhibits or delays the formation of critical components of fish innate immunity, preventing antiviral or proinflammatory responses (Abram et al. 2017).

Recent studies have examined the importance of dietary approaches in reducing the negative influences of thermal stress on various fish species (Behairy et al. 2023; Alfons et al. 2023; Refaey et al. 2022). Herbiotics effectively promote growth, manage health, and enhance the immune system of fish (Abu-Zahra et al. 2024; El-Gammal et al. 2025). *Pelargonium sidoides* (PS) extract is one of the most significant herbs utilized in traditional medicine by the native inhabitants of southern Africa (Moyo and Van Staden 2014). In traditional medicine, PS roots are used for the treatment of infectious respiratory disorders, such as tuberculosis (Gajewski et al. 2021). According to Moyo and Van Staden (2014), the main components are coumarins and polymeric polyphenols, and most coumarins have a methoxy group, which endows them with antibacterial properties. There is a significant amount of gallic acid and its methyl ester, which are the main immunostimulatory components of this herbal remedy (Moyo and Van Staden 2014). Furthermore, PS contains many phytochemicals, amino acids, minerals, and vitamins that improve bodily functions and guard against disease (Kolodziej et al. 2003).

Understanding the relationship between temperature anomalies and the thermal tolerance of *O. niloticus* is crucial for developing effective management practices in aquaculture. This study hypothesized that dietary supplementation with PS extract alleviates the oxidative stress and immune suppression induced by extreme temperatures (17 °C, 25 °C, and 33 °C) in *O. niloticus*. By measuring antioxidant enzyme activities, immunological parameters, pathological deviations, and disease resistance, this study systematically evaluated the efficacy of PS extract in mitigating thermal stress. To the best of our knowledge, this study is the first to determine the effects of PS extract on the survival rate and growth of *O. niloticus* reared under thermal stress.

## Materials and methods

### Estimation of the total phenolic and total flavonoid contents in the PS (ethanolic extract)

The ethanolic extract of PS roots was obtained from Marcyrl Pharmaceutical Industries Company, Egypt. Each 1 ml (1.0261 gm) contained 0.833 ml of PS root extract (equivalent to 0.823 gm/density 0.988 gm/ml). The solvent was 11% (w/w) ethanol, and the mixture was dried. The total phenolic and flavonoid contents of the extracts were measured colorimetrically, and the absorbance was measured at 765 and 510 nm against the reagent blank following the methods of Sembiring et al. (2017) and Marinova et al. (2005), respectively. The results are expressed as milligrams of gallic acid equivalent (GAE) and quercetin equivalent (QUE) per 1 g of dry extract for total phenolic and flavonoid contents, respectively.

### Evaluation of the antioxidant activity of PS via the DPPH radical scavenging method

The 1,1-diphenyl-2-picryl hydrazyl (DPPH) technique of González-Palma et al. (2016) was used to determine the plant root extract’s capacity to scavenge free radicals. The absorbance was determined at 517 nm via a spectrophotometer (UV‒VIS, Milton Roy). The experiment was carried out in triplicate, and ascorbic acid was employed as a reference standard. The log dose inhibition curve was used to calculate the sample’s IC_50_ value, the concentration needed to inhibit 50% of DPPH-free radicals. The lower absorbance of the reaction mixture suggested higher levels of free radical activity. To calculate DPPH scavenging %, or percent inhibition, the following formula was used:

$$\:{\%}\:\text{i}\text{n}\text{h}\text{i}\text{b}\text{i}\text{t}\text{i}\text{o}\text{n}=\frac{\text{A}1-\text{A}2}{\text{A}1}\times\:100$$

where A2 is the absorbance when the test or standard sample is present, and A1 is the absorbance of the control reaction.

### In vitro antimicrobial activity of PS

The antibacterial activity of PS against *A. hydrophila* was detected via the agar well diffusion method as described by Al-Sagheer et al. (2017). The bacterial strain was previously isolated from clinically affected *O. niloticus* and identified biochemically and molecularly (El-Gammal et al. 2025). The strains were cultured for 24 h at 37 °C in Tryptic Soy Agar. Mueller–Hinton broth was used to cultivate pure cultures of the bacterial strains for 24 h at 37 °C. Using McFarland standards, the bacterial suspension was adjusted to a bacterial cell density of 1 × 10^7^ CFU/ml. The entire surface of the Mueller‒Hinton agar plates was inoculated with a sterile swab dipped in the bacterial suspension. Using gel puncture, wells with a diameter of 6 mm were made on MHA plates. Each well of all the plates received approximately 30 µl of diluted PS at different concentrations (0, 5, 10, 20, and 30 µl/mL) in dimethyl sulfoxide (DMSO). Positive controls were treated with the antibiotic levofloxacin (10 µg/mL). The plates were then incubated at 37 °C for a full day. The diameter of the inhibition zones (mm) was determined. All plates were carried out in triplicate.

### Diet preparation

Two diets, a control (CTR) diet without any additives and a PS diet in which the CTR diet was enriched with 3% PS/kg diet, were made. The CTR diet was purchased from Aller-Aqua Co., Egypt. The raw materials of the commercial CTR diet listed on the label are DDGS (distiller-dried grain soluble), corn gluten, fish oil, fish meal, rice products, maize, soya, soy oil, sunflower meal, wheat products, yeast, vitamins and minerals. The CTR diet contained (based on the information provided on the producer label) 36% crude protein, 6% crude fat, 7.7% ash, 5.1% crude fat, 1% phosphorous, 37.2% nitrogen-free extract, 18.1 MJ gross energy, and 10.2% digestible energy. The liquid PS extract was well mixed with a finely powdered commercial diet in which water (450 mL/kg) was added to create a uniform paste. The diets were extruded through an electric meat mincer (2 mm diameter), air-dried, and stored at 4 °C. The CTR diet was also prepared in the same manner.

### Fish rearing conditions and experimental design

*O. niloticus* (*n* = 270 with a mean weight of 57.69 ± 1.05 g) were acquired from a local fish farm at the Kafrelsheikh governorate, Egypt. The fish were kept in 18 glass aquaria (50 × 40 × 40 cm) that contained unchlorinated tap water. The fish were fed the CTR diet for 14 days before the trial began (at a water temperature of 25 ± 1 °C). The fish were haphazardly dispersed into 6 triplicate groups (*n* = 45 fish/group, 15 fish/replicate). On the basis of previous studies on various freshwater fish species, including *Clarias gariepınus* (Turan 2018), our preliminary trial, and our results concerning the in vitro antibacterial activity of PS against *A. hydrophila*, the dietary level of PS was selected. This study was conducted in February 2024 during the winter season, when the water temperature was below 17 °C throughout the duration of the experiment. The heaters were used to adjust the water temperature to 17 °C, 25 °C, and 33 °C for all the groups. The water temperature increased from 25 to 33 °C or decreased from 25 to 17 °C within four days (by 2 °C per day) to protect the fish from thermal shock before the official trial. Half of the water was exchanged daily with preheated water (17, 25, and 33 °C), and the debris was removed. Fish behaviors were detected throughout the exposure time (30 days). The water parameters, including temperature, ammonia (NH3), dissolved oxygen (DO), and pH, were checked daily throughout the trial. The temperature and pH were monitored with a digital waterproof pHep^®^ pH/temperature tester (HI98128; Hanna Instruments Inc., RI, USA), and the NH3 concentration was determined via a spectrophotometric method. The DO concentrations were measured via a portable dissolved oxygen meter (using the Standard Polarographic DO Probe -HI76407-Hanna Instruments Inc.). Each parameter was recorded at the same time every day to ensure consistency. The recorded values were within the acceptable limits: NH3 = 0.02 ± 0.002 mg/L, DO = 6.50 ± 0.25 mg/L, and pH = 7.4 ± 0.2.

Each group received their respective diets until satiation twice daily at 8:30 am and 13:30 pm for 6 days/week. The fish were considered satisfied when they rejected the feed, and the feed intake was estimated for each tank. Table 1 illustrates the design of the experiment.

**Table 1:** Experimental design of *O. niloticus* groups subjected to thermal stress (TS) for 30 days and/or fed diets containing PS root extract

Fish groups	Code	No. fish	Water temperature	Pelargonium sidoides roots extract (PS)
1	T25 (CTR)	45	25 °C	×
2	T25 + PS	45	25 °C	30 mL/kg feed
3	T33	45	33 °C	×
4	T33 + PS	45	33 °C	30 mL/kg feed
5	T17	45	17 °C	×
6	T17 + PS	45	17 °C	30 mL/kg feed

T25 (CTR): fish fed a CTR diet without additives at 25 °C, T25 + PS (PS group): fish fed a CTR diet enriched with 3% PS at 25 °C, T33 (heat-stressed group): fish fed a CTR diet without additives at 33 °C, T33 + PS: fish fed a CTR diet enriched with 3% PS at 33 °C, T17 (cold stress group): fish fed a CTR diet at 17 °C, T17 + PS group: fish fed a CTR diet enriched with 3% PS at 17 °C

### Calculation of growth indices

As stated by Abu-Zahra et al. (2024), the following equations were used to calculate the growth indices:

$$\:\text{W}\text{G}\:\left(\text{g}\right)=\text{F}\text{W}\:\left(\text{g}\right)-\text{I}\text{W}\:\left(\text{g}\right)$$

$$\:\text{G}\text{a}\text{i}\text{n}\:{\%}=\frac{\text{W}\text{G}}{\text{I}\text{W}}\times\:100$$

$$\:\text{S}\text{G}\text{R}=\frac{\text{ln}\text{F}\text{W}-\text{ln}\text{I}\text{W}}{\:\text{D}\text{a}\text{y}\text{s}\:\text{o}\text{f}\:\text{e}\text{x}\text{p}\text{e}\text{r}\text{i}\text{m}\text{e}\text{n}\text{t}}\times\:100$$

$$\:\text{F}\text{C}\text{R}=\frac{\text{T}\text{F}\text{I}}{\text{W}\text{G}}$$

$$\:\text{F}\text{E}=\frac{\text{W}\text{G}}{\text{T}\text{F}\text{I}}$$

$$\:\text{M}\text{R}\:{\%}=\frac{\:\text{N}\text{u}\text{m}\text{b}\text{e}\text{r}\:\text{o}\text{f}\:\text{d}\text{e}\text{a}\text{d}\:\text{f}\text{i}\text{s}\text{h}\:\:\:}{\text{T}\text{o}\text{t}\text{a}\text{l}\:\text{n}\text{u}\text{m}\text{b}\text{e}\text{r}\:\text{o}\text{f}\:\text{f}\text{i}\text{s}\text{h}\:}\times\:100$$

where WG: weight gain, FW: final weight, IW: initial weight, SGR: specific growth rate, FCR: feed conversion ratio, TFI: total feed intake, FE: feed efficiency, and MR: mortality rate.

### Blood and tissue sampling

When the experiment was finished, the fish were starved for 24 h. Three fish/aquarium (*n* = 9/group) were collected and sedated for approximately 5 min with 100 mg/L benzocaine (Behairy et al. 2023) to decrease handling stress. Then, 3 mL syringes were used to draw blood from the caudal veins. Blood was collected for serum purposes without the use of an anticoagulant, and it was allowed to coagulate at room temperature before being centrifuged at 3000 rpm for 15 min. The clear sera were subsequently separated and stored at −20 °C until biochemical analysis. For estimation of hematological parameters, nitroblue tetrazolium, and phagocytosis, another set of blood samples was collected via a 1-mL heparinized syringe. The pH of the blood samples used in our study was 7.5 ± 0.1. The fish were subsequently dissected (*n* = 9/group), and their livers, gills, and intestines were removed and preserved in 10% formalin for histopathological study.

### Hematological analyses

Following the protocol of Yılmaz and Ergün (2018), an auto hematology analyzer (DH36, Shenzhen Dymind Biotechnology Co.) was used to measure red blood cells (RBCs), hematocrit (Ht), hemoglobin (Hb), mean corpuscular volume (MCV), mean corpuscular hemoglobin (MCH), mean corpuscular hemoglobin concentration (MCHC), and total and differential leukocyte counts (WBCs).

### Serum biochemical analysis and antioxidant parameters

Commercial reagent kits from SPINREACT Co., Spain, were used to detect the serum levels of lactate dehydrogenase (LDH), aspartate aminotransferase (AST), alanine aminotransferase (ALT), urea, creatinine, triglyceride (TG), cholesterol (CHO), high-density lipoprotein (HDL), and low-density lipoprotein (LDL) via an ARX-199 chemical analyzer (Micro Lab Instrument, India) following the manufacturer’s instructions. Using glucose GOD-POD kits acquired from SPINREACT Co., Spain, and an ELISA commercial kit from MyBioSource Inc., USA, the serum glucose (Trinder 1969) and cortisol hormone levels were measured.

The levels of glutathione peroxidase (GPx), superoxide dismutase (SOD), and catalase (CAT) were estimated via diagnostic ELISA kits (Cusabio Biotech Co., Ltd.; China) following the manufacturer’s guidelines. Malondialdehyde (MDA) levels were measured via the technique of Uchiyama and Mihara (1978) and reported as nmol/mL. Details on the kits used and the serum biochemical and antioxidant marker data are presented in Table 1S.

### Immune parameters

Total protein and albumin were estimated according to Doumas et al. (1981), while globulin levels were estimated mathematically. The serum lysosomal activity was determined via a turbidimetric assay following the methods of Ellis (1990), which were based on the lysis of *Micrococcus lysodeikticus* (Sigma, USA). In brief, 66 mM phosphate buffer (pH 6.0) was used to prepare a standard solution of 0.15 mg/mL *M. lysodeikticus*. A spectrophotometer was used to record the absorbance reduction at 450 nm at 30-s and 4.5-min intervals after adding 50 µL of serum to 1 mL of the bacterial culture. Lyophilized chicken egg white lysosomes (Sigma Co., USA) were serially diluted to form a calibration curve to estimate the serum lysosome content. A 0.001/min decrease in absorbance indicates one unit of lysosome. Phagocyte activity (PA%) and index (PI no.) were determined via heat-inactivated *Candida albicans*, and the subsequent formula followed Kawahara et al. (1991):

$$\:\text{P}\text{A}{\%}=\frac{\text{N}\text{u}\text{m}\text{b}\text{e}\text{r}\:\text{o}\text{f}\:\text{p}\text{h}\text{a}\text{g}\text{o}\text{c}\text{y}\text{t}\text{e}\text{s}\:\text{w}\text{i}\text{t}\text{h}\:\text{e}\text{n}\text{g}\text{u}\text{l}\text{f}\text{e}\text{d}\:\text{b}\text{a}\text{c}\text{t}\text{e}\text{r}\text{i}\text{a}}{\text{N}\text{u}\text{m}\text{b}\text{e}\text{r}\:\text{o}\text{f}\:\text{p}\text{h}\text{a}\text{g}\text{o}\text{c}\text{y}\text{t}\text{e}\text{s}\:}\times\:100$$

$$\:\text{P}\text{I}\:\text{N}\text{o}=\frac{\text{N}\text{u}\text{m}\text{b}\text{e}\text{r}\:\text{o}\text{f}\:\text{e}\text{n}\text{g}\text{u}\text{l}\text{f}\text{e}\text{d}\:\text{b}\text{a}\text{c}\text{t}\text{e}\text{r}\text{i}\text{a}}{\text{N}\text{u}\text{m}\text{b}\text{e}\text{r}\:\text{o}\text{f}\:\text{p}\text{h}\text{a}\text{g}\text{o}\text{c}\text{y}\text{t}\text{e}\text{s}}\times\:100$$

Serum bactericidal activity (SBA) was estimated via the procedures of Biller-Takahashi et al. (2013a). After a 24-h incubation period, the colonies from the resulting incubated mixture were counted in triplicate on TSA plates (3 plates/sample) to determine the number of live bacteria. A decreased bacterial count is correlated with improved serum bactericidal activity. Total serum immunoglobulin (TIg) was evaluated following the method described by Siwicki et al. (1994). Myeloperoxidase (MP) activity was evaluated following the methods of Yilmaz et al. (2021). A total of 90 µL of HBSS solution was used to dilute a 10 µL serum sample. 3,3′,5,5′-Tetramethylbenzidine dihydrochloride solution and hydrogen peroxide (H₂O₂) were subsequently added to this mixture. The reactions were stopped with 35 µL H_2_SO_4_ after two minutes, and measurements were taken at 450 nm and 24 °C via a Multiscan microplate reader.

Nitroblue tetrazolium (the respiratory burst of leukocytes) was estimated according to the methods of Biller-Takahashi et al. (2013b). The technique uses nitroblue tetrazolium dye to determine reactive oxygen species colorimetrically. At 540 nm, the optical density of the solution was measured with a spectrophotometer.

### Histological examination

The histomorphology was performed according to Suvarna et al. (2019). Samples of the intestine, gills, and liver (*n* = 9/group) were chopped into 0.5 cm³ portions, which were then preserved for 24 h in 10% formalin. Following a series of alcohol dehydration steps, the samples were cleaned with xylene and embedded in paraffin wax. After that, hematoxylin and eosin (H&E) staining was used to stain 5 μm thick sections, which were cut with a Leica rotatory microtome (Leica Microsystems, Germany). Finally, the tissue sections were examined via a light microscope (Leica DM 5000) connected with a digital camera. The histological scoring of nine X200 power fields, each covering an area of approximately 11–12 mm^2^, was performed within the hepatopancreas and gills. Additionally, in accordance with the guidelines of Wilson et al. (2018), quantitative measurements of the intestinal villus length (from tip to base) and width and goblet cell number were performed on 9 complete, full-sized villi that did not exhibit any mechanical damage, bending, or fixation. The ImageJ analysis program (National Institutes of Health, MD, USA) was used for all measurements of the intestinal villi. The results were then reported in micrometers.

### Experimental infection

A virulent *A. hydrophila* strain that had been previously isolated from *O. niloticus* and molecularly characterized (El-Gammal et al. 2025) was experimentally injected intraperitoneally into 30 fish/group (10 fish/replicate) at the end of the trial (only 180 fish were selected for experimental challenge). The bacterial isolate was incubated for 24 h at 37 °C after being subcultured in TSB. The concentration of the bacterial suspensions was adjusted to 1 × 10^7^ CFU/ml via McFarland standard tubes. A total of 0.2 mL of bacterial suspension was injected into each fish via the procedures of Ezzat et al. (2018). The clinical signs and mortality rate were recorded daily for 10 days.

### Statistical analysis

Prior to analysis, the data were tested via the Kolmogorov‒Smirnov and Levene tests to ensure normality and homogeneity of variance. Two-way ANOVA was used to analyze all the data, and Tukey’s test was run at the 95% significance level (*P* ≤ 0.05). In accordance with the damage to the respective tissues from their normal structure, the main histological alterations in the gills and hepatopancreas were identified and quantitatively assessed via a seven-point ordinal scale. Using pairwise comparisons and a nonparametric Kruskal‒Wallis test, the quantitative scores of the lesions (means ± SEs) were assessed. SPSS software version 22 for Windows was used to conduct the statistical analysis. Microsoft Excel 2016 and SPSS software version 22 were used to draw the figures.

## Results

### Total phenolic and total flavonoid contents and antioxidant activity of PS roots

**Fig. 1:**
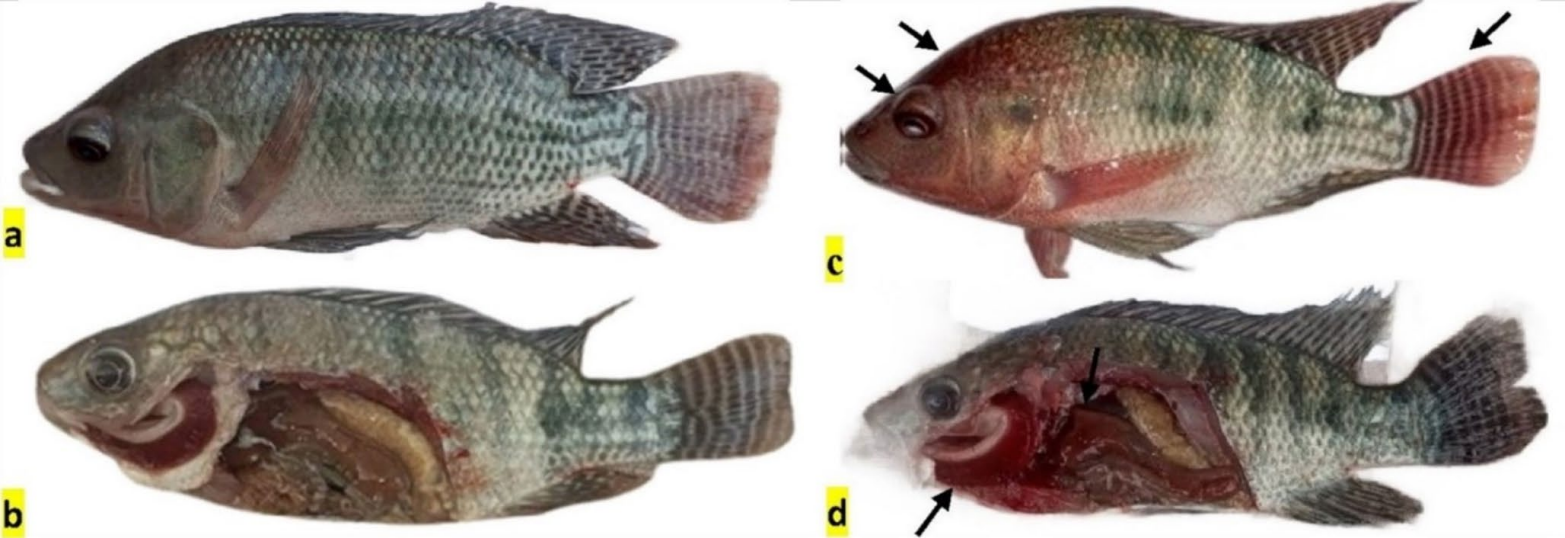
Clinical and postmortem examination of *O. niloticus* in the experimental groups subjected to thermal stress (TS) and/or fed diets enriched with PS for 30 days. (**a**) T25 (CTR): showing normal appearance; (b) T17: showing normal appearance; (**c**) T33: showing diffuse hemorrhage on the body surface and fins, ocular hemorrhage, and exophthalmia; (**d**) T33: showing congested gills and hemorrhagic liver

**Fig. 2:**
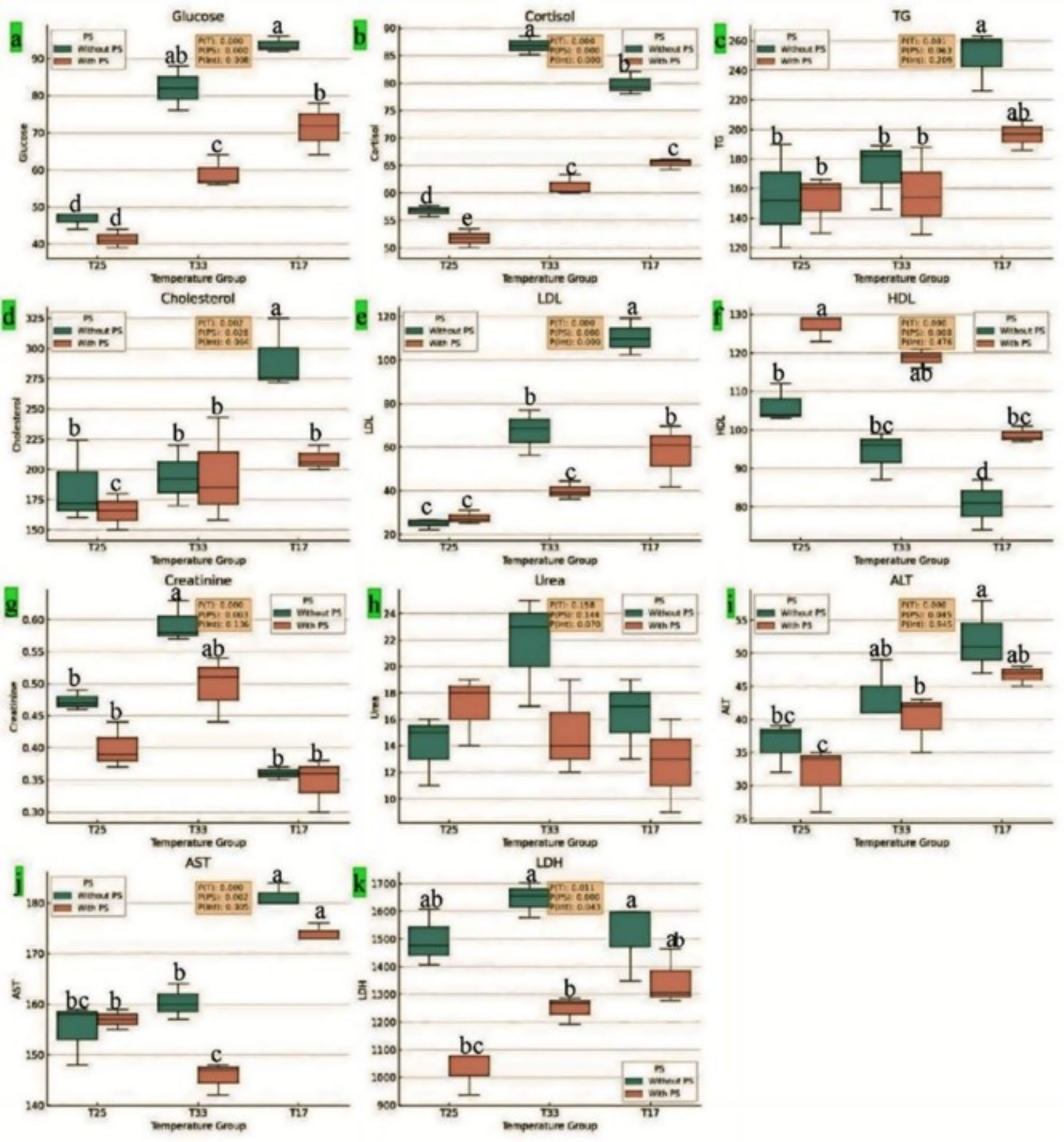
Effects of dietary PS on stress and biochemical parameters in *O. niloticus* exposed to thermal stress (TS) for 30 days. (**a**) glucose (mg/dL), (**b**) cortisol (ng/L), (**c**) TG (mg/dL): triglycerides, (**d**) total cholesterol (mg/dL), (**e**) LDL (mg/dL): low-density lipoprotein, (**f**) HDL (mg/dL): high-density lipoprotein, (**g**) creatinine (mg/dL), (**h**) urea (mg/dL), (**i**) ALT (U/L): alanine aminotransferase, (**j**) AST (U/L): aspartate aminotransferase, **k**) LDH (U/L): lactate dehydrogenase. The black line inside each box represents the median (50th percentile) of the data distribution for that group, indicating the middle value when the data are ordered from smallest to largest. The box (interquartile range, IQR) contains the middle 50% of the data. The lower edge of the box represents the 25th percentile (Q1), and the upper edge of the box represents the 75th percentile (Q3). The error bars (whiskers) extend to the smallest and largest values within 1.5 × IQR from the first and third quartiles, indicating the spread of the data beyond the IQR. *n* = 9/group, and the different subscripts above the bars indicate significant differences between treatments (*P* < 0.05)

The total phenolic and total flavonoid contents of the PS root extracts were 27.7 ± 0.03 mg GAE (gallic acid)/g dry extract and 66.1 ± 0.18 mg QUE (quercetin)/g dry extract, respectively (Table 2S and Fig. 1S). The antioxidant activity of the PS root extracts was 0.971 ± 0.001 µg TE/ml, the DPPH scavenging percentage was 39.7%, and the IC_50_ was 5.5 µg/ml (Table 2S, Table 3S, and Fig. 2S). The results were dose dependent.

### In vitro antibacterial activity of PS against *A. hydrophila*

The various tested PS concentrations (5, 10, 20, and 30 µl/mL) strongly inhibited the isolated pathogenic *A. hydrophila*, as indicated in Table 2. There was an increase in the diameter of the inhibitory zone, which was concentration dependent. The 30 µl/mL PS treatment had the greatest effect on *A. hydrophila*.

**Table 2:** In vitro antibacterial activity of PS against *A. hydrophila*

Antibacterial agent	Inhibition zone (mm)
PS (µl/mL)	
0 (-ve Control)	0.0
5	10.4 ± 0.23^e^
10	14.5 ± 0.05^d^
20	17.2 ± 0.09^c^
30	24.1 ± 0.09^b^
+ Control (antibiotic)	
Levofloxacin (10 µg/mL)	26.3 ± 0.15^a^

The values are the means ± SEs (*n* = 3). At *P* ≤ 0.05, various letters indicate significant differences

### General observations of behavior and clinical signs

The fish kept at 25 °C and fed either a CTR diet (T25, CTR) or a diet supplemented with PS (T25 + PS) presented a normal appetite and swimming behavior throughout the feeding trial (30 days). On the other hand, the fish in the groups reared at 17 °C and fed the CTR diet (T17) presented reduced swimming behavior, anorexia, and lethargy. However, those reared at 33 °C (T33) and fed the CTR diet experienced increased ventilation rates, swimming toward the water surface, and dark coloration. Conversely, the fish reared at either 33 °C or 17 °C and fed a PS-supplemented diet (T33 + PS or T17 + PS) presented a relatively normal appetite and swimming behavior.

Clinical and postmortem examinations revealed that fish exposed to heat stress (T33) presented diffuse hemorrhage on the body surface and fins, ocular hemorrhage, exophthalmia, congested gills and hemorrhagic liver (Fig. 1c, d), whereas those exposed to the same heat stress and fed PS (T33 + PS) presented dark coloration, some skin hemorrhage, and a normal appearance of the internal organs. However, the fish exposed to cold stress presented a normal appearance (Fig. 1b).

### Growth performance

Thermal stress and PS supplementation significantly affected growth performance metrics such as FW, WG, G%, TFI, FCR, FE, and SGR (Table 3). The T25 + PS group consistently presented superior growth performance and feed efficiency across various metrics. PS supplementation was beneficial for enhancing growth performance and feed efficiency under both optimal (25 °C) and stress (17 °C and 33 °C) conditions. Compared with those of the CTR fish, notable decreases in FW, WG, G%, TFI, and SGR values were documented in the fish subjected to cold water stress at 17 °C (T17) for 30 days. The heat-stressed group (33 °C, T33) presented significantly lower growth and efficiency parameters, with greater FCRs and lower FEs. Remarkably, thermal stress combined with dietary PS (T33 + PS and T17 + PS) elicited substantial and similar improvements in growth parameters compared with those of the thermal stress groups (T33 and T17) and were similar to those of the CTR group. Throughout the experiment, no mortalities were observed in any of the groups.

**Table 3:** Effects of dietary PS on the growth indices and survival rates of *O. niloticus* subjected to thermal stress (TS) for 30 days

Parameter	T25 (CTR)	T25 + PS	T33	T33 + PS	T17	T17 + PS	Two-way ANOVA (*P*- value)		
							Temperature	PS	Interaction
IW (g)	58.27 ± 0.76	57.52 ± 0.19	58.68 ± 0.49	57.71 ± 0.25	57.29 ± 0.24	56.67 ± 0.43	0.221 (ns)	0.110	0.532 (ns)
FW (g)	73.56 ± 0.84^b^	80.20 ± 1.03^a^	69.15 ± 0.97^c^	75.56 ± 1.06^b^	65.67 ± 0.79^c^	72.30 ± 0.73^b^	0.001***	0.001***	0.001***
WG (g)	15.28 ± 0.76^c^	22.68 ± 0.90^a^	10.47 ± 0.78^d^	17.85 ± 0.96^b^	8.38 ± 0.14^d^	15.63 ± 0.19^c^	0.001***	0.001***	0.002**
Gain %	26.30 ± 1.23^c^	39.45 ± 1.87^a^	17.90 ± 1.16^d^	30.97 ± 1.89^b^	14.63 ± 1.02^e^	27.58 ± 1.51^c^	0.001***	0.001***	0.001***
TFI (g)	18.75 ± 0.06^a^	16.26 ± 0.04^b^	18.42 ± 0.06^a^	18.25 ± 0.06^a^	10.27 ± 0.11^d^	13.53 ± 0.10^c^	0.001***	0.001***	0.002**
FCR	1.25 ± 0.02^b^	0.72 ± 0.01^d^	1.82 ± 0.04^a^	1.03 ± 0.02^c^	1.23 ± 0.02^b^	0.87 ± 0.01^d^	0.001***	0.001***	0.002**
FE	0.81 ± 0.02^c^	1.39 ± 0.03^a^	0.57 ± 0.01^d^	0.98 ± 0.02^b^	0.81 ± 0.02^c^	1.16 ± 0.02^b^	0.001***	0.001***	0.001***
SGR %	0.18 ± 0.01^b^	0.26 ± 0.01^a^	0.13 ± 0.01^d^	0.21 ± 0.01^b^	0.11 ± 0.01^d^	0.19 ± 0.01^b^	0.001***	0.001***	0.003**
SR %	100	100	100	100	100	100	-	-	-

Significant differences are observed between the means ± SEs in the same row with different letters (*n* = 9/group). The asterisks above the *P* values indicate the level of statistical significance in the ANOVA results. ns: not significant (*P* > 0.05), *: significant (*P* ≤ 0.05), **: highly significant (*P* ≤ 0.01), ***: very highly significant (*P* ≤ 0.001)

### Hematological parameters

Thermal stress significantly affects RBC count, Hb, Ht, and WBCs, with the most pronounced effect observed under cold stress (T17). The exposure of the fish to cold stress induced significant decreases in RBC count, Hb level, and Ht %, along with reduced WBC, LYM, and GRA counts. In contrast, PS supplementation (T25 + PS, T33 + PS, and T17 + PS) improved RBC counts and Hb levels, especially under high-temperature stress (T33 + PS) (Table 4). The protective effect of PS was evident under both optimal and stress conditions. The hematological parameters of the fish exposed to heat stress without PS supplementation did not significantly change. These results demonstrate that different temperatures and PS have significant impacts on various blood parameters, with notable changes in RBC, Hb, Ht, and leukogram (WBC and LYM counts), among other parameters. The interaction between temperature and PS also yielded significant effects on several parameters, highlighting the complex interplay of these factors with respect to blood characteristics.

**Table 4:** Effects of dietary PS on hematological parameters in *O. niloticus* subjected to thermal stress (TS) for 30 days

Parameter	T25 (CTR)	T25 + PS	T33	T33 + PS	T17	T17 + PS	Two-way ANOVA (*P*- value)		
							Temperature	PS	Interaction
Erythrogram									
RBCs (×10^6^/mm^3^)	1.95 ± 0.05^c^	2.25 ± 0.05^b^	2.03 ± 0.12^c^	2.94 ± 0.06^a^	1.62 ± 0.05^d^	2.10 ± 0.08^c^	0.001***	0.001***	0.003**
Hb (g/dl)	9.90 ± 0.29^c^	12.77 ± 0.25^a^	10.30 ± 0.21^b^	12.57 ± 0.57^a^	7.17 ± 0.31^d^	10.03 ± 0.30^bc^	0.002**	0.001***	0.010*
Ht (%)	33.50 ± 0.66^c^	38.60 ± 0.25^b^	33.33 ± 0.73^c^	44.33 ± 1.47^a^	25.10 ± 1.29^d^	27.63 ± 2.28^d^	0.001**	0.001***	0.004**
MCV (fl.)	168.50 ± 4.97^b^	163.30 ± 6.82^c^	175.07 ± 5.04^a^	170.17 ± 7.43^b^	162.17 ± 6.08^c^	157.03 ± 5.39^d^	0.050*	0.003**	0.075 (ns)
MCH %	50.37 ± 2.56^a^	51.30 ± 1.73^a^	50.60 ± 2.82^a^	43.90 ± 2.52^b^	50.63 ± 2.34^a^	48.17 ± 3.31^ab^	0.004**	0.030*	0.150 (ns)
MCHC %	30.43 ± 1.05^bc^	31.43 ± 0.51^a^	28.86 ± 1.34^c^	28.15 ± 1.67^c^	30.13 ± 1.32^bc^	36.96 ± 2.91^a^	0.010*	0.015*	0.050*
RDW-CV%	15.73 ± 0.69^bc^	15.17 ± 0.55^c^	17.67 ± 0.81^ab^	16.23 ± 0.92^bc^	17.57 ± 0.91^ab^	16.43 ± 0.72^bc^	0.080 (ns)	0.045*	0.200 (ns)
Leukogram									
WBCs (×10^3^/mm^3^)	56.24 ± 1.09^bc^	59.77 ± 0.91^ab^	58.36 ± 1.18^ab^	59.93 ± 0.71^a^	51.14 ± 0.85^c^	61.86 ± 1.26^a^	0.001***	0.001***	0.004**
LYM (×10^3^/mm^3^)	48.95 ± 0.95^b^	52.51 ± 0.88^a^	50.76 ± 1.10^ab^	52.75 ± 0.85^a^	46.90 ± 0.91^c^	56.96 ± 1.10^a^	0.002**	0.001***	0.010*
MID (×10^3^/mm^3^)	5.50 ± 0.20^ab^	3.59 ± 0.15^bc^	4.46 ± 0.25^ab^	3.78 ± 0.24^bc^	3.72 ± 0.20^bc^	4.04 ± 0.24^ab^	0.040*	0.030*	0.100 (ns)
GRA (×10^3^/mm^3^)	2.46 ± 0.31^b^	4.00 ± 0.41^a^	2.82 ± 0.25^ab^	3.39 ± 0.34^ab^	0.98 ± 0.21^c^	0.86 ± 0.13^c^	0.001***	0.001***	0.005**

Significant differences are observed between the means ± SEs in the same row with different letters (*n* = 9/group). The asterisks above the *P* values indicate the level of statistical significance in the ANOVA results. ns: not significant (*P* > 0.05), *: significant (*P* ≤ 0.05), **: highly significant (*P* ≤ 0.01), ***: very highly significant (*P* ≤ 0.001). RDW: red cell distribution width; LYM: lymphocytic count; MID: total count of white blood cells that are not categorized as granulocytes or lymphocytes; GRA: granulocytes

### Stress biomarkers and biochemical parameters

Thermal stress significantly impacted all the measured parameters, with dietary PS providing varying degrees of mitigation. The interplay between temperature and dietary PS underscores the importance of dietary interventions for managing stress and associated biochemical changes in *O. niloticus*. Compared with the T25 (CTR) group, the T25 + PS group presented a noticeable reduction in the serum cortisol level and an insignificant reduction in the glucose level. Both glucose and cortisol were significantly higher in the TS groups (T33 and T17) than in the T25 group (CTR) (*P* < 0.05) (Fig. 2). However, the inclusion of PS in the diets of the TS groups significantly reduced stress biomarkers compared with those in the TS groups, which were fed a CTR diet, with values near those of the T25 (CTR) group. Dietary inclusion of 3% PS at T25°C did not significantly change hepatorenal function (ALT, LDH, and creatinine) or triglyceride levels but significantly decreased cholesterol and increased HDL levels (*P* < 0.05) in fish reared at 25 °C. However, there was a tendency toward an increase in liver enzyme levels and lipid profiles, except for HDL, which notably decreased after the exposure of the fish to cold water stress (T17). In contrast, exposure to heat stress (T33) did not significantly (*P* > 0.05) impact most of these parameters except for creatinine, LDH enzyme and LDL, which significantly increased. Compared with those in the TS groups (T33 and T17), significant improvements in the serum biochemical parameters were found in the TS + PS groups, with values approaching those of the T25 (CTR) group.

### Immune parameters and oxidative/antioxidant balance

**Fig. 3:**
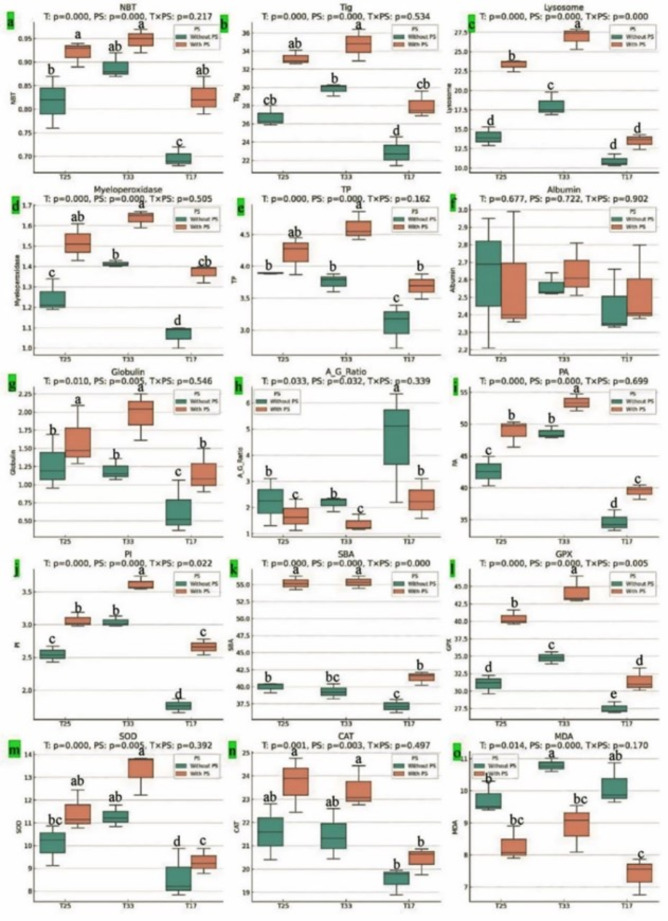
Effects of dietary PS on the oxidative/antioxidative status and immune parameters of *O. niloticus* subjected to thermal stress (TS) for 30 days. (**a**) NBT (mg/mL): nitroblue tetrazolium, (**b**) TIg (µg/mL): total immune globulin, (**c**) lysosomal activity (mg/mL), (**d**) MP (OD 450 nm): myeloperoxidase, (**e**) TP (g/dL): total serum protein, (**f**) albumin (g/dL), (**g**) globulin (g/dL), (**h**) A/G ratio, (**i**) PA%: phagocytic activity, (**j**) PI No: phagocytic index, **k**) SBA %: serum bactericidal activity, **l**) GPx (U/mL): glutathione peroxidase, **m**) SOD (U/mL): superoxide dismutase, **n**) CAT (U/mL): catalase, **o**) MDA (nmol/mL): malondialdehyde. The black line inside each box represents the median (50th percentile) of the data distribution for that group, indicating the middle value when the data are ordered from smallest to largest. The box (interquartile range, IQR) contains the middle 50% of the data. The lower edge of the box represents the 25th percentile (Q1), and the upper edge of the box represents the 75th percentile (Q3). The error bars (whiskers) extend to the smallest and largest values within 1.5 × IQR from the first and third quartiles, indicating the spread of the data beyond the IQR. *n* = 9/group, and the different subscripts above the bars indicate significant differences between treatments (*P* < 0.05)

Compared with the CTR diet, the 3% PS diet significantly increased immune parameters, such as phagocytic activity and index (PA and PI), serum bactericidal activity (SBA), nitroblue tetrazolium (NBT), lysosome, and MP activity (Fig. 3). The exposure of *O. niloticus* to cold water stress induced immune suppression through decreases in total protein (TP) content; phagocytosis (PA and PI); SBA; and the levels of nitrobenzene blue (NBT), myeloperoxidase (MP), and total immunoglobulin (Tig). In contrast, the exposure of fish to heat stress caused significant increases in phagocytosis, MP, and lysosomal activity. A tendency toward an increase in all immune parameters (*P* < 0.05) was recorded in the TS + PS (T33 + PS and T17 + PS) groups compared with the TS (T33 and T17) groups.

The levels of SOD and GPx were significantly reduced by cold stress exposure at 17 °C, with insignificant decreases in CAT (*P* > 0.05). The serum MDA levels were higher in the fish exposed to heat stress. Compared with those in the TS groups, dietary PS (T33 + PS and T17 + PS) significantly (*P* < 0.05) improved the changes in the serum antioxidant and MDA levels induced by thermal stress. Overall, the dietary addition of PS extract significantly mitigated the immune and antioxidant parameters of *O. niloticus* subjected to thermal stress, helping to maintain fish health under adverse temperature conditions.

### Histopathological examination

**Fig. 4:**
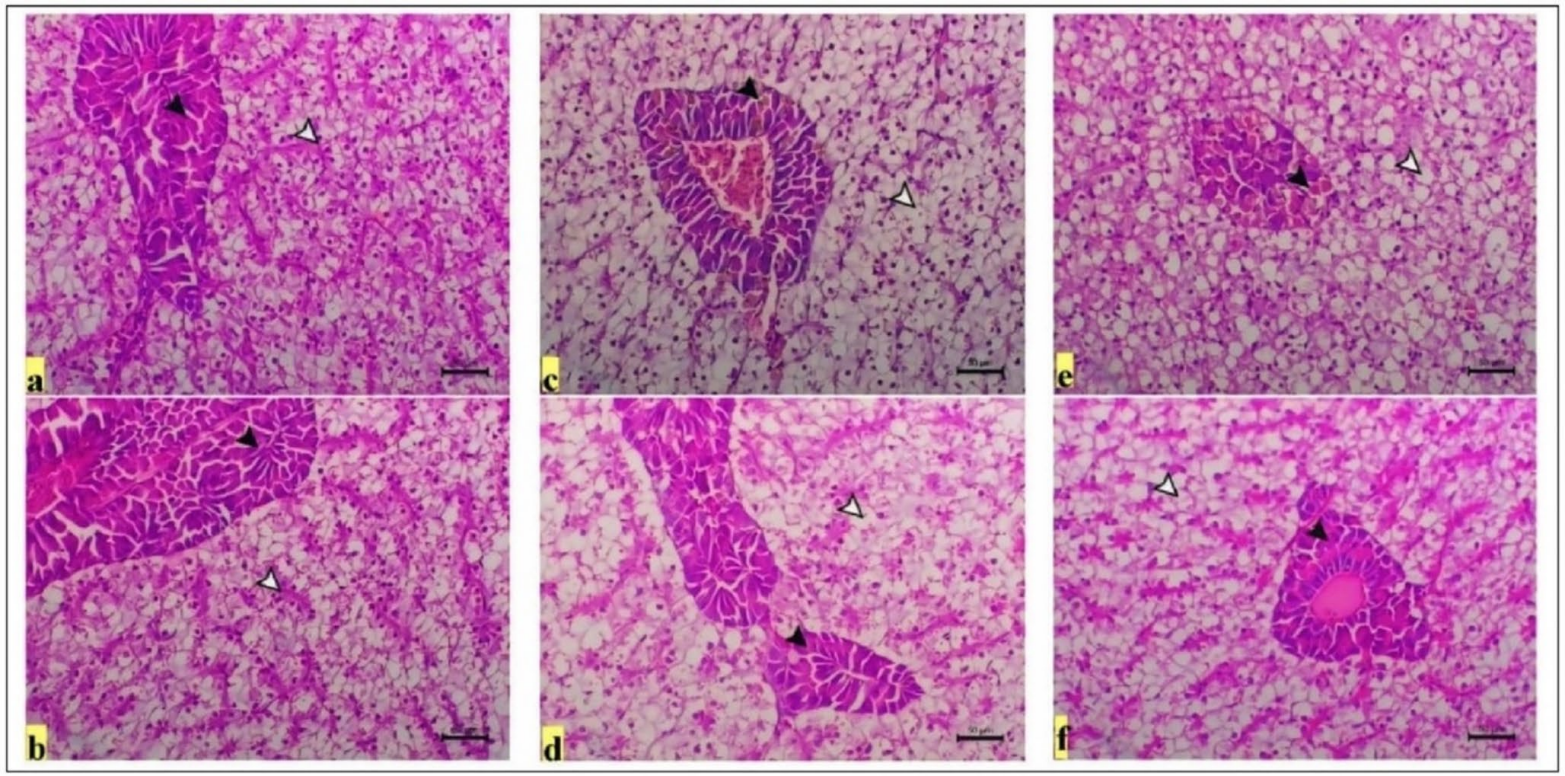
Representative photomicrographs of H&E-stained hepatopancreases of *O. niloticus* in the experimental groups subjected to thermal stress (TS) and/or fed diets enriched with PS for 30 days. Scale bar = 50 μm. (**a**) T25 (CTR) showing normal hepatic cells with mild hepatic vacuolation consistent with fat storage (white arrowhead) and with the pancreatic portion around the central vein (black arrowhead), (**b**) T25 + PS showing normal hepatic (white arrowhead) and pancreatic portions (black arrowhead), (**c**) T33 showing marked hepatic vacuolation associated with nuclear pyknosis (white arrowhead) with infiltration of melanomacrophage cells within the pancreatic portion (black arrowhead), (**d**) T33 + PS showing a remarkable decrease in hepatic vacuolation (arrowhead) and with normal pancreatic portions (arrow), (**e**) T17 showing marked hepatic degenerative changes associated with severe hepatic vacuolation (white arrowhead) and marked infiltration of melanomacrophage cells within the pancreatic portion (black arrowhead), (**f**) T17 + PS showing a marked decrease in hepatic vacuolation (white arrowhead) and with normal pancreatic portions (black arrowhead)

The histological alterations in the hepatopancreas and gills were quantitatively assessed (Table 5). Histopathological examination of T25 (CTR) revealed normal hepatic cells with mild hepatic vacuolation consistent with fat storage and with a normal pancreatic portion around the central vein (Fig. 4a). A similar normal histological structure was also observed in the hepatopancreas of the T25 + PS group but without hepatic vacuolation (Fig. 4b; Table 5). However, in the thermally stressed groups, marked hepatic degenerative changes (T17) associated with severe (T17) to marked (T33) hepatic vacuolation and nuclear pyknosis (T33) with infiltration of melanomacrophage cells within the pancreatic portion (Fig. 4c and e) were reported. This disrupted tissue damage was partially restored when PS was included in the fish diets (T33 + PS and T17 + PS), where the fish presented a marked decrease in hepatic vacuolation and normal pancreatic portions (Fig. 4d, f).

**Table 5:** Quantitative evaluation of the major histological changes in the *O. niloticus* groups

Histological changes	*Quanitative evaluation on a seven point scale					
	T25 (CTR)	T25 + PS	T33	T33 + PS	T17	T17 + PS
Hepatopancreas						
Hepatic vacuolation	3.27 ± 0.08^c^	1.25 ± 0.16^e^	5.45 ± 0.05^b^	2.05 ± 0.13^d^	6.80 ± 0.70^a^	2.67 ± 0.10^d^
Nuclear pyknosis	0.68 ± 0.09^d^	0.45 ± 0.08^d^	5.56 ± 0.08^a^	1.19 ± 0.12^c^	2.24 ± 0.04^b^	1.75 ± 0.75^c^
Infiltration of MMC	0.36 ± 0.10^c^	0.42 ± 0.04^c^	5.68 ± 0.12^a^	1.91 ± 0.14^b^	5.70 ± 0.19^a^	1.24 ± 0.081^b^
Hepatic degeneration	0.82 ± 0.08^c^	0.75 ± 0.18^c^	1.23 ± 0.20^b^	0.50 ± 0.11^c^	5.31 ± 0.08^a^	0.22 ± 0.04^c^
Gills						
↑length of SL	1.27 ± 0.07^b^	5.04 ± 0.18^a^	1.05 ± 0.83^b^	1.91 ± 0.11^b^	1.18 ± 0.06^b^	1.56 ± 0.17^b^
Atrophy	0.75 ± 0.75^e^	0.00 ± 0.00^e^	5.43 ± 0.14^a^	2.27 ± 0.07^c^	3.27 ± 0.09^b^	1.21 ± 0.07^d^
Necrosis	0.31 ± 0.08^d^	0.05 ± 0.01^d^	5.18 ± 0.08^a^	2.24 ± 0.08^b^	2.31 ± 0.10^b^	1.31 ± 0.10^c^
Adhesion of SL	1.23 ± 0.20^C^	0.00 ± 0.00^d^	5.52 ± 0.07^a^	3.28 ± 0.04^b^	5.21 ± 0.07^a^	3.70 ± 0.19^b^
Congestion of the FC	0.22 ± 0.04^d^	0.25 ± 0.83^d^	3.68 ± 0.12^b^	1.43 ± 0.14^c^	5.96 ± 0.10^a^	3.35 ± 0.08^b^
Thickening of SL	1.18 ± 0.08^c^	0.21 ± 0.11^d^	4.15 ± 0.16^a^	3.24 ± 0.04^b^	4.04 ± 0.08^a^	3.00 ± 1.04^b^
ICI	0.18 ± 0.06^d^	0.28 ± 0.04^d^	2.91 ± 0.14^b^	1.56 ± 0.17^c^	5.52 ± 0.16^a^	2.52 ± 0.16^b^

*Ordinal scale for quantitative evaluation: 0: no change, 1: normal (< 5% of tissues affected), 2: decreased (5–15% of tissues affected), 3: mild (15–25% of tissues affected), 4: moderate (25–50%), 5: marked (50–75%), and 6: severe (> 75%). The quantitative evaluation was based on nine observations for each organ of the particular group. Significant differences (*P* < 0.05) are observed between the means ± SEs in the same row with different letters (*n* = 9/group). MMC: melanomacrophage cells, SL: secondary lamellae, FC: filamentous capillaries, ICI: inflammatory cell infiltration

**Fig. 5:**
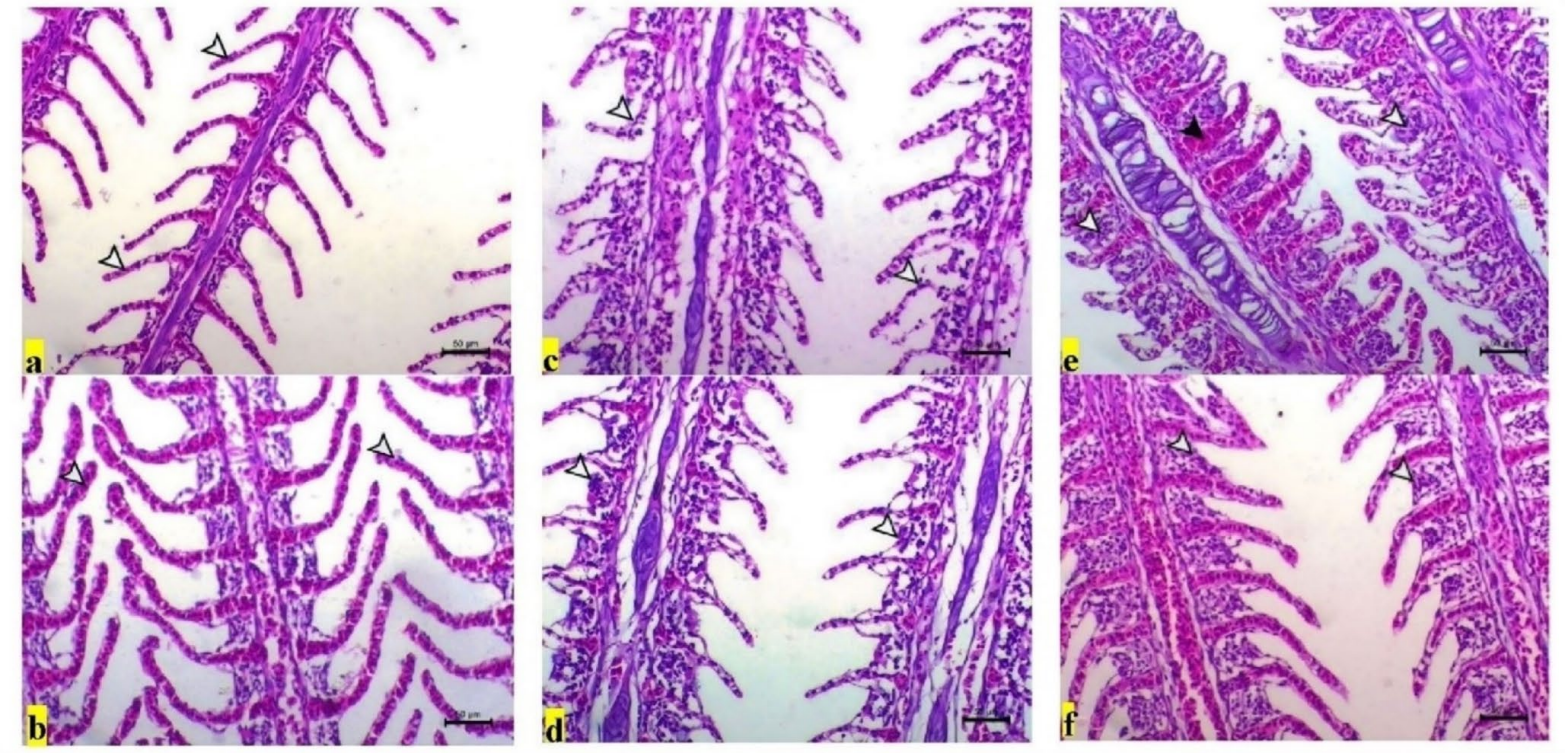
Representative photomicrographs of H&E-stained gill tissue from *O. niloticus* in the experimental groups subjected to thermal stress (TS) and/or fed diets enriched with PS for 30 days. Scale bar = 50 μm. (**a**) T25 (CTR) showing normal secondary lamellae (arrowheads), (**b**) T25 + PS showing normal long secondary lamellae with markedly increased length (arrowheads), (**c**) T33 showing atrophy, necrosis and adhesion of the secondary lamellae (arrowheads), (**d**) T33 + PS showing mild thickening and adhesion of the secondary lamellae (arrowheads), (**e**) T17 showing congestion of the filamentous capillaries (black arrowhead) and marked adhesion of the secondary lamellae with marked inflammatory cell infiltration (white arrowheads), and (**f**) T17 + PS showing mild thickening and adhesion of the secondary lamellae (arrowheads)

Like those of the hepatopancreas, the gill tissue of the T25 (CTR) group had a normal structure with normal secondary lamellae (Fig. 5a; Table 5). However, the gill tissue of the T25 + PS group presented normal long secondary lamellae with a marked increase in length (Fig. 5b). A clear picture of gill damage, including atrophy, necrosis, adhesion of the secondary lamellae (T33, Fig. 5c), congestion of the filamentous capillaries and marked adhesion of the secondary lamellae associated with marked inflammatory cell infiltration (T17, Fig. 5e), was observed in the fish groups subjected to thermal stress (T33 and T17). Again, the gills of the fish in the groups subjected to thermal stress and fed diets enriched with PS showed signs of improvement, with mild thickening and adhesion of the secondary lamellae (Fig. 5d, f).

**Fig. 6:**
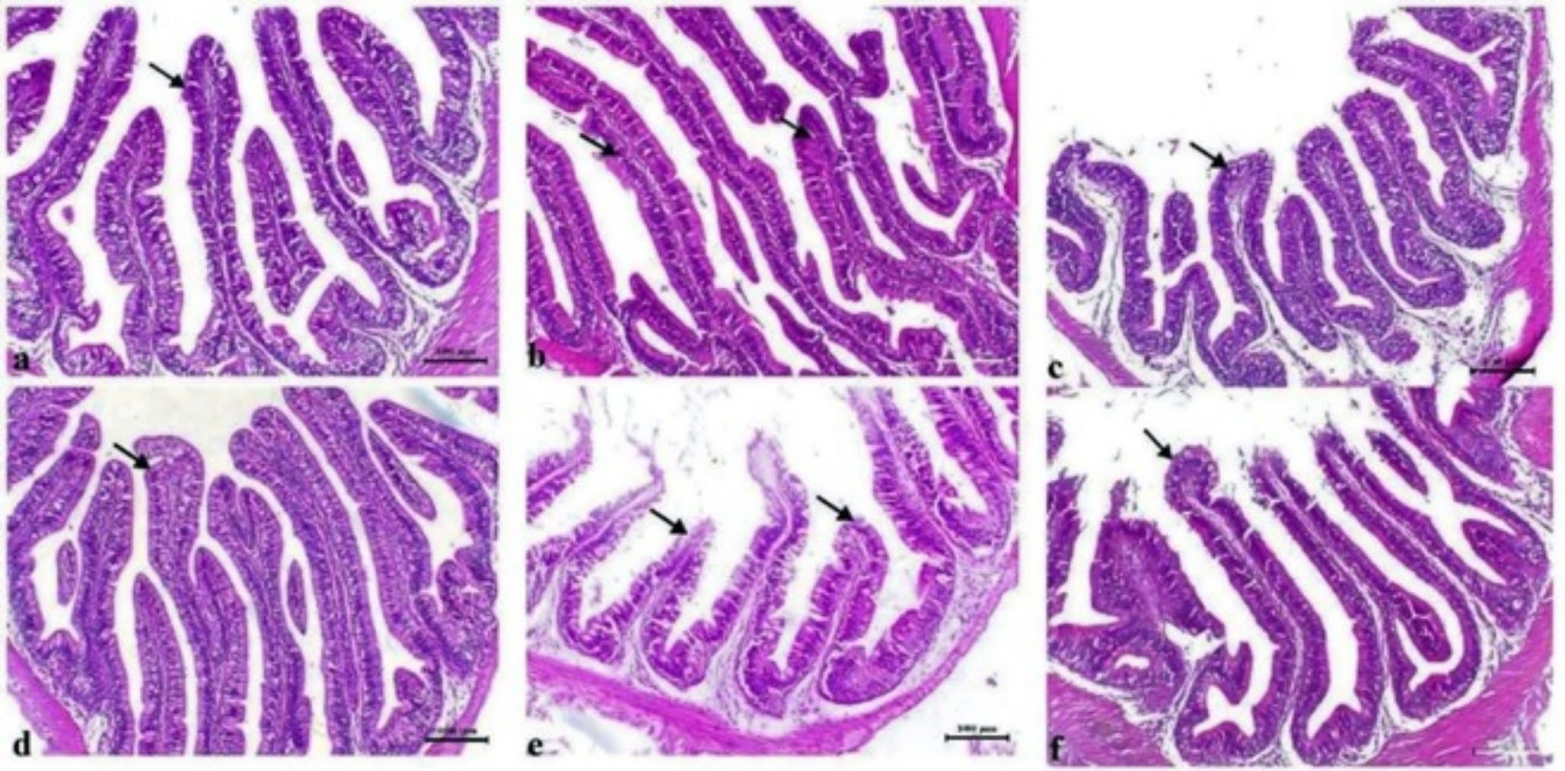
Representative photomicrograph of H&E-stained intestines (middle portion) and histomorphometry of *O. niloticus* in the experimental groups subjected to thermal stress (TS) and/or fed diets supplemented with PS for 30 days, X100, scale bar = 100 μm. (**a**) T25 (CTR) showing normal intestinal villi lined with pseudostratified epithelium with goblet cells (arrow), (**b**) T25 + PS showing a marked increase in intestinal villus length (arrows), (**c**) T33 showing a remarkable decrease in intestinal villus length (arrow), (**d**) T33 + PS showing an increase in intestinal villus length (arrow), (**e**) T17 showing a marked decrease in intestinal villus length with an increase in the space between it (arrows), and (**f**) T17 + PS showing an increase in villus length (arrow)

The intestinal tissue (middle portion) of the T25 (CTR) group presented a normal structure with normal intestinal villi lined with pseudostratified epithelium with goblet cells (Fig. 6a). The intestinal villi of the T25 + PS group markedly increased in length (Fig. 6b). Like in the hepatopancreas and gills, a clear picture of intestinal damage, including a remarkable decrease in intestinal villus length (Fig. 6c, e) with increasing space between villi (Fig. 6e), was recorded in the fish groups subjected to thermal stress (T33 and T17). The intestinal tissue of the fish subjected to thermal stress and fed diets containing PS improved with increasing villus length (Fig. 6d, f).

**Fig. 7:**
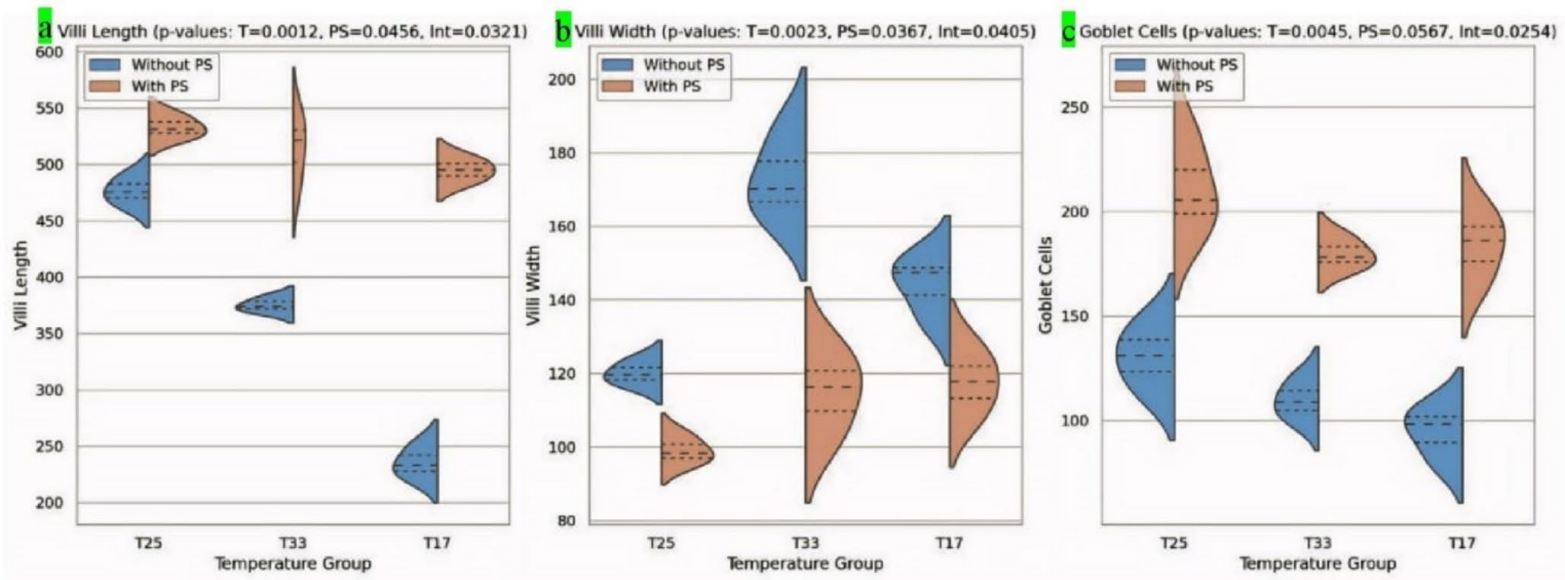
Violin plots showing the distributions of (**a**) villi length (µm), (**b**) villi width (µm), and (**c**) goblet cell number (per mm²) in the middle portion of the intestinal tissue of *O. niloticus* subjected to thermal stress (TS) for 30 days and fed diets with or without PS. Each plot illustrates the data spread and quartiles, with blue indicating groups without PS and orange representing groups with PS. The mean and quartile ranges are marked within each violin. The results of a two-way ANOVA for the effects of temperature (T), PS supplementation (PS), and their interaction (T × PS) are presented, with significant *P* values displayed in the figure

The results of the intestinal morphometric analysis are shown in Fig. 7. Compared with T25 (CTR), thermal stress caused a significant reduction in villus length with an insignificant decrease in the number of goblet cells, especially in the cold-stressed group (T17). The groups that were subjected to thermal stress presented the greatest variation in villus width, suggesting the most severe form of disruption (Fig. 7). PS notably mitigated the adverse effects of thermal stress on the villi length and number of goblet cells (T33 + PS and T17 + PS), with values approaching those of the CTR group at T25.

### Experimental challenge

**Fig. 8:**
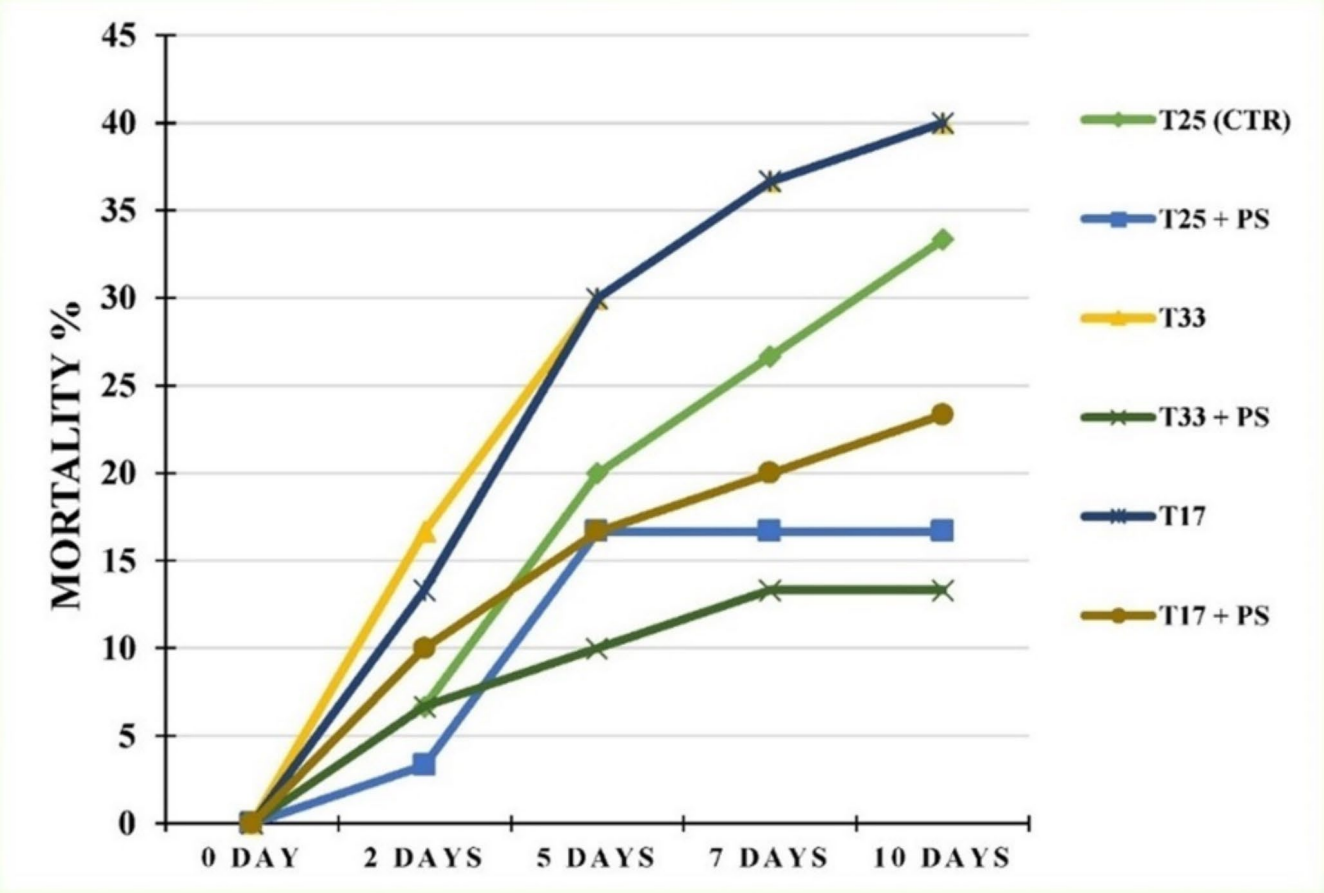
Effects of the experimental treatments on the mortality rates of *O. niloticus* (*n* = 30/group) after 10 days of challenge with *A. hydrophila*

The cumulative mortalities of the fish groups after 10 days of *A. hydrophila* challenge are shown in Fig. 8. Compared with those in the other treatment groups, the mortality rates in the PS-supplemented groups, even those under thermal stress, were lower. The lowest mortality rates were recorded in the T33 + PS (13.33%) and T25 + PS (16.67%) groups, followed by the T17 + PS (23.33%) group, whereas the highest mortality rates were recorded in the thermally stressed groups (T33 and T17, 40%) compared with the T25 (CTR, 33.33%) group.

**Fig. 9:**
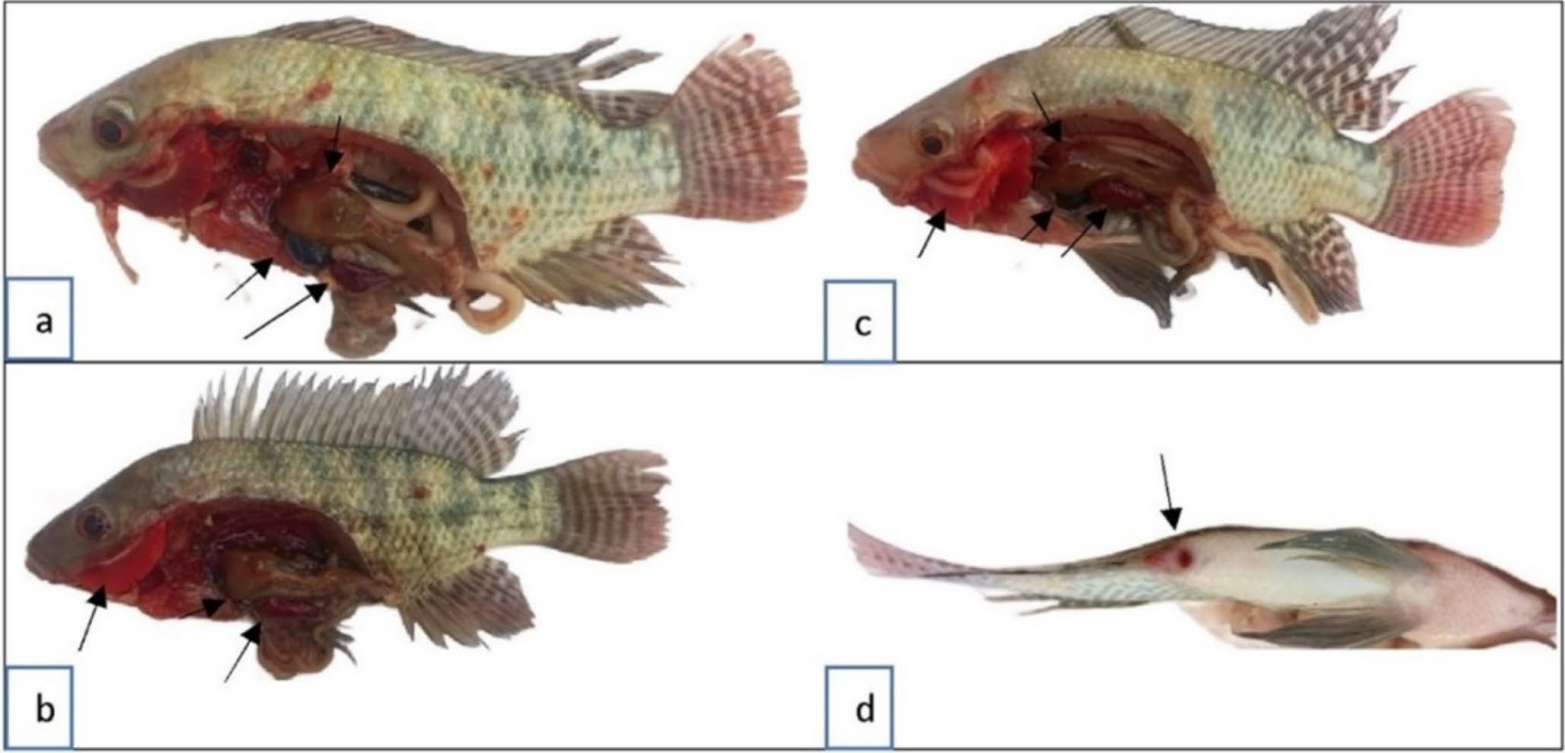
Clinical and postmortem examination of *O. niloticus* groups after *A. hydrophila* challenge. The arrows for each graph indicate the observed signs. (**a**) T25 (CTR) showing green (retention of bile in the liver) hemorrhagic liver, splenomegaly, and distended gall bladder; (**b**) T33 showing congestion of the internal organs and gills, hemorrhagic spots on the liver, and splenomegaly; (**c**) T33 + PS showing hemorrhagic spots on the liver, congested gills, splenomegaly, and distended gall bladder; (**d**) T17 + PS showing hemorrhagic anus and genital papilla

Clinical and postmortem examination of the *O. niloticus* groups after *A. hydrophila* challenge (Fig. 9; Table 6) revealed ocular opacity and exophthalmia, pale heart, ascites, congestion of the internal organs and gills, hemorrhagic spots on the liver, splenomegaly, distended gall bladder, retention of bile in the liver, hemorrhagic anus and genital papilla. The signs were more severe in the fish exposed to thermal stress (TS).

**Table 6:** Clinical and postmortem examination of *O. niloticus* groups after *A. hydrophila* challenge

Item	T25 (CTR)	T25 + PS	T33	T33 + PS	T17	T17 + PS
Ocular opacity	-	-	-	-	++	-
Exophthalmia	-	-	+	-	++	-
Ascites	+	-	+	-	++	-
Dark skin	+	-	-	-	++	-
Hemorrhagic liver	+	+	++	+	++	+
Retention of bile in the liver	++	-	++	-	++	-
Distended gall bladder	++	+	+++	+	+	+
Splenomegaly	+	+	+++	+	+	+
Congested gills	-	-	+	+	-	-
Pale heart	++	-	-	-	++	-
Hemorrhagic anus/genital papilla	-	-	-	-	-	+

+++: Severe, ++: Moderate, +: Mild, and -: Absent (no lesions)

## Discussion

Our study revealed that thermal stress (33 °C and 17 °C for 30 days) significantly affects the growth, hematology, biochemistry, immunity, and histological structure of fish. Additionally, the use of phytogenic compounds, such as PS, in fish diets has gained interest (Abu-Zahra et al. 2024; El-Gammal et al. 2025), but PS application in *O. niloticus* culture is not well documented.

### Phytogenic compounds

Our results revealed that the primary constituents of PS, including phenols (27.7 ± 0.03 mg GAE/g dry extract) and flavonoids (66.1 ± 0.18 mg QUE/g dry extract), exhibit strong antibacterial properties. Furthermore, geranium (*Pelargonium graveolens*) and other floral-related essential oils have been shown to exhibit strong antimicrobial properties against a range of microbes (Al-Sagheer et al. 2017). These effects are primarily due to a combination of several biologically active compounds, including linalool, isomenthone, citronellol, and many of their derivatives (Al-Sagheer et al. 2017). More recently, it was shown that the antibacterial action of *Pelargonium* may also be aided by components that are present in relatively small amounts, such as caryophyllene, rose oxide, pinene, and linalool (Nadjib Boukhatem et al. 2013).

### General observations of behavior and clinical signs

The fish reared at 17 °C exhibited reduced swimming behavior and lethargy, whereas those reared at 33 °C exhibited increased ventilation rates and dark coloration. However, fish fed a PS-enriched diet displayed a normal appetite and swimming behavior, even under thermal stress. The heat-stressed fish presented symptoms such as hemorrhage and exophthalmia, whereas the PS-fed fish presented fewer severe symptoms.

In agreement with our observations, Behairy et al. (2023) reported several behavioral changes in *O. niloticus* subjected to cold stress (18 °C for 2 months). These authors attributed these changes to the marked decrease in AChE (acetylcholinesterase) activity. Oxidative stress can negatively impact fish by causing cellular damage (Lee et al. 2020), which may lead to altered behavior. Studies have shown that antioxidants, such as those present in dietary PS, can help mitigate oxidative stress and improve overall health and behavior in fish. For example, antioxidants can protect neurons from oxidative damage, resulting in improved neural function (Lee et al. 2020) and improved behavioral responses. We noticed general signs of body exhaustion triggered by the exposed temperature levels. Similarly, Zutshi et al. (2020) noted a deviated swimming pattern in juvenile Koi carp reared at 35 °C, whereas at 15 °C, the fish became sluggish and spent much of their time at the tank bottom. Additionally, fish exhibit a shift in body and gill color and scale loss in both temperature ranges (Zutshi et al. 2020).

### Dietary PS improves growth performance

The growth performance of the fish exposed to thermal stress at 17 °C and 33 °C substantially decreased. Adding PS to the diets of thermally stressed fish significantly improved growth performance, which aligns with findings that PS enhances fish growth under stress conditions.

Growth rates typically increase with temperature; however, at very low or high temperatures, organisms either die or become dormant (Li et al. 2023). There is usually a threshold temperature above which growth is inhibited. For example, *Coreius guichenoti* can grow and survive at temperatures ranging from 14 to 30 °C, while its growth performance is greatest at 26.55–28.33 °C (Li et al. 2023). In line with our findings, *Lateolabrax maculatus* kept at 27 °C presented significantly greater growth performance than did those kept at 33 °C (Cheng et al. 2021). These findings also demonstrated that one of *O. niloticus*’s most important environmental factors is water temperature. Fish grow more quickly when the water temperature is high (as long as it is within the optimum range), since this increases feed intake and metabolic rates (Li et al. 2023). However, when water temperatures rise above the optimum range, FE and SGR are likely to decline with increasing feed intake, which decreases over time (Li et al. 2023). The reduced growth performance observed in this study is consistent with that reported by Refaey et al. (2023), who attributed the decline to the fact that fish under cold stress utilize stored energy to endure extreme conditions. This forces the body to use the energy it has stored, which results in weight reduction (Refaey et al. 2023).

Additionally, the results of the present study revealed no adverse effects of the PS extract-based diet on *O. niloticus* survival, feed intake or FCR, which were substantially improved in the experimental groups fed PS-based diets even under thermal stress. The in vitro inhibition of *A. hydrophila* showed that PS has high antibacterial activity, which may be strongly associated with its growth-enhancing impact. Therefore, the antibacterial properties of PS and the pharmacological characteristics of its active ingredients may have positive impacts on fish performance.

### Dietary PS enhances hematological indices

In the present study, cold stress caused significant microcytic and hypochromic anemia. According to Barros et al. (2009), cold stress hinders erythropoiesis, which lowers the capacity of an organism’s tissue to transport oxygen. As a result, fish are unable to maintain the minimum quantity of red blood cells necessary to maintain their health. Furthermore, cold stress causes metabolism to return to homeostasis rather than conserve energy, which hinders the body from obtaining enough nutrients for the optimum formation of Hb (Behairy et al. 2023). Furthermore, leukopenia may occur because circulation slows during cold stress, which hinders the release of leukocytes from the myeloid sinuses into the bloodstream (Behairy et al. 2023). Conversely, the findings of the present study revealed that adding PS to the diet reduced the impacts of cold stress on hematological changes. Our results revealed that, compared with those of T25 (CTR), the hematological parameters of fish exposed to heat stress did not significantly differ.

### Dietary PS reestablishes the disturbed tissue function triggered by thermal stress

In the present study, fish reared under thermal stress and fed a basal diet may have greater serum cortisol and glucose levels because of their thermal adaptability to a lower enzymatic reaction rate (Dellagostin et al. 2022). Additionally, it can indicate a greater need for energy to counteract the negative consequences of stress. According to Rotllant et al. (2001), the pituitary‒interrenal axis of *Sparus aurata* is altered by a decrease in water temperature, which results in the release of cortisol. It has been shown that stressed fish have higher plasma glucose levels because of the increased metabolic requirements supplied by gluconeogenesis and the breakdown of glycogen into glucose (Khieokhajonkhet et al. 2022; Islam et al. 2019). Several fish species, such as *P. pangasianodon* and hybrid catfish (Khieokhajonkhet et al. 2022; Islam et al. 2019), have been reported to exhibit elevated levels of glucose and cortisol following extreme thermal exposure. On the other hand, dietary PS also significantly decreased the negative effects of thermal stress on blood cortisol and glucose levels in *O. niloticus*.

On the basis of the findings of this study, fish under cold stress presented significantly increased levels of total cholesterol and triglycerides, which suggests that they use more energy to address repair processes (Behairy et al. 2023). In *Lateolabrax maculatus*, an increase in total cholesterol, which serves as a vital energy source, has been observed under cold stress (Wang et al. 2022). Conversely, fish that were fed a diet supplemented with PS presented decreased lipid synthesis and increased lipid metabolism.

According to our study, elevated liver enzyme activity at both high and low temperatures points to the utilization of free amino acids for energy synthesis. Alfons et al. (2023) reported comparable findings in *Clarias gariepinus* as a result of heat stress. However, the dietary inclusion of PS significantly improved the liver function of the thermally stressed fish. This may be due to the flavonoid compounds in PS, which are known for their antioxidant, anti-inflammatory, and hepatoprotective effects.

### Dietary PS ameliorates immune responses

Notably, the total serum protein concentration decreases dramatically in fish raised at low temperatures, suggesting that the activation of protein catabolism induces immunological dysfunction (Islam et al. 2020) and skeletal muscle degradation and reduces or stops growth until or unless safety precautions are taken, which puts the fish at risk of death. Lysosomes react to changes in water temperature. In this context, the exposure of the fish to heat stress in this study caused significant increases in lysosomal activity.

The most prevalent proinflammatory enzyme found in the azurophilic granules of neutrophilic granulocytes is myeloperoxidase (MP). Increased MP levels have been linked to excessive ROS generated by heat-induced oxidative stress. This study revealed that granulocytes were stimulated by heat stress and the addition of PS to the diet. After the administration of immunostimulants to fish, several authors reported an increase in MP activity (Awad et al. 2013; Yılmaz and Ergün 2018).

In *O. niloticus* reared under thermal stress and fed PS-supplemented diets, the immunomodulatory effect of PS may have improved immune parameters. Similarly, the PA, PI, SBA, and NBT contents and lysosomal activity significantly increased in the PS group, possibly because PS supplementation improved the immune status. Elevated serum protein profiles are associated with weight gain and improved liver function, which is the organ responsible for protein synthesis. The increase in total protein levels following PS administration suggested improvements in protein digestion and absorption, increased effectiveness of protein utilization, and increased weight gain. Our findings revealed that the PS extract had greater levels of flavonoids and phenolic compounds with strong antioxidant activity. Nevertheless, which of the active components are responsible for the impacts on immune function that have been noted is unclear. The improvement in general health may be the cause of the increased innate immunity of these fish.

### Dietary PS restores the oxidative/antioxidant balance

According to the present findings, cold stress significantly reduced SOD activity and GPx activity but did not significantly decrease CAT activity. According to several recent studies, cold stress significantly compromised the antioxidant capacity of fish, which might have altered the quantity and activity of both antioxidant enzymes and MDA-related enzymes (Refaey et al. 2023). PS inclusion at 30 mL/kg did not significantly increase the antioxidant activity of the fish stored at 25 °C and restored the alterations in SOD and GPx activity triggered by cold stress at 17 °C. These findings indicate that PS can replenish endogenous antioxidants, scavenge excess ROS, and increase antioxidant enzyme activity to protect against cold stress-induced oxidative stress. According to Al-Sagheer et al. (2017), O. *niloticus* fed diets containing *Pelargonium graveolens* presented increased antioxidant capacity, which could be attributed to its capacity to lower ROS generation.

Under thermal stress (17 °C and 33 °C), the MDA content increased, and similar results were reported in *Cyprinus carpio var koi* (Zutshi et al. 2020). High temperatures generally disrupt lipid stability over time and increase the possibility of free radical production during the breakdown of fatty acids (Zutshi et al. 2020).

### Dietary PS repairs the disturbed tissue structure triggered by thermal stress

The liver performs essential physiological activities such as excretion, detoxification, and metabolism, and an organism’s liver state can most accurately reflect the body’s nutritional physiology and pathological condition (Alfons et al. 2023). According to an earlier study, thermal stress can alter the structure and even function of the liver (Alfons et al. 2023). In the present study, thermal stress caused the liver to undergo histological and ultrastructural changes, as well as hepatic degenerative changes associated with severe to marked hepatic vacuolation and nuclear pyknosis with infiltration of monocytic cells within the pancreas. According to our study, PS has hepatoprotective effects. These findings corroborate those of prior studies showing that adding *Pelargonium* to fish diets improved metabolism and enhanced physiological performance and the immune response in *Clarias gariepınus* (Turan 2018) and *O. niloticus* (Al-Sagheer et al. 2017). The hepatic vacuolation observed in the CTR group may be related to seasonal variations (Shimadzu et al. 2013), as the study was performed during the winter season.

The gills are among the most vital organs in fish. Since fish gills are in close proximity to the aquatic environment, they are subjected primarily to stressors in the water (Perry and Laurent 1993). In this study, the PS group reared at 25 °C presented normal long secondary lamellae with a marked increase in length. Remarkable gill damage, including atrophy, necrosis, and adhesion of the secondary lamellae, was detected in the fish subjected to thermal stress. In contrast, the gills of the fish in the groups subjected to thermal stress and fed diets containing PS improved, with mild thickening and adhesion of the secondary lamellae. Phrompanya et al. (2021) detected anomalies such as aneurysms in the gills of fish exposed to heat shock. An aneurysm is indicated by the collection of blood in the secondary lamellae. The aberrant characteristic in question may be the consequence of increased blood flow and hemorrhage caused by a breakdown in vascular integrity and a rupture of the pillar cell system. These differences might be due to *O. niloticus* being subjected to higher temperatures (37 °C) than those used in our study (33 °C), in addition to the rapid increase in water temperature (3 °C/h). Gill atrophy, necrosis, and adhesion of secondary lamellae may increase the diffusion distance and slow gas exchange activities (Phrompanya et al. 2021). These findings suggest that thermal stress reduces the ability of the gills to exchange gases, which hinders the intake of oxygen. Under cold temperature, congestion of the filamentous capillaries and marked adhesion of the secondary lamellae associated with marked inflammatory cells were recorded in our study. These changes may constitute a protective strategy for gill tissue to reduce the surface area exposed to thermal stress from the surrounding environment. These changes generally lengthen the gap between the blood and the external environment and act as barriers to the entry of pollutants (Phrompanya et al. 2021).

Notable alterations in the morphometric properties of the middle intestinal portion in the thermal stressed groups were also observed. Indeed, a clear picture of intestinal damage, including a remarkable decrease in intestinal villus length with increasing space between villi, was recorded in the fish groups subjected to thermal stress. Similarly, earlier studies have shown that the structure of the intestinal villi, especially epithelial cells, substantially changes under heat stress in various fish species, such as *O. mykiss* and *C. carpio* (Zhao et al. 2024; Dawood et al. 2022). The innate and adaptive immunity of fish may be further impacted by intestinal damage, which could result in inflammatory responses (Zhao et al. 2024).

### Dietary PS enhances the survival rate after *A. hydrophila* infection

In this study, the survival rate of *O. niloticus* decreased to 60% within 10 days after being challenged with *A. hydrophila* under thermal stress. Previous studies have demonstrated that this pathogen can enter fish through the intestine and settle in organs such as the kidney, spleen, liver, and brain (Zhao et al. 2024). Thus, it may be concluded that the intestinal damage triggered by thermal stress renders *A. hydrophila* vulnerable to invasion, increasing the risk of *O. niloticus* infection and death. As shown in previous studies, the mortality rate of *O. mykiss* reached 100% after 24 h of challenge with *A. salmonicida* under heat stress (Zhao et al. 2024). These results collectively revealed that fish under thermal stress presented decreased resistance to infections. Importantly, even under thermal stress, the PS-supplemented groups presented higher survival rates than did the other treatment groups post challenge, which may be related to the antimicrobial activity of PS and the enhanced immune and antioxidant status. The lowest survival was recorded in the thermally stressed groups fed the basal diet, which was related to the depressed immunity, oxidative imbalance, and pathological changes observed here. It is clear that dietary PS may possess antistress properties, as indicated by a decrease in blood glucose and cortisol levels and increased antioxidant activity.

## Conclusion

This study revealed that thermal stress, especially cold stress at 17 °C, causes oxidative stress, immune suppression, and histological aberrations in *O. niloticus*. Nonetheless, the dietary addition of PS (30 mL/kg diet) has the potential to improve fish growth, immunity, antioxidant capacity, and general health while also lowering histological aberrations following thermal stress. Because PS has immunomodulatory and antioxidant properties, it may be a safe and eco-friendly feed supplement to diminish the negative effects triggered by thermal stress in fish. However, further mechanistic studies and validation are needed to confirm these findings. The results of the present study revealed that dietary PS extract at a concentration of 30 mL/kg is effective under the current experimental conditions for *O. niloticus*. We anticipate that the results of this study will motivate further studies on the use of PS in *O. niloticus* diets.

### Limitations and future research

In our study, we did not include sham injection controls. There is a need for future studies to incorporate sham controls to ensure a more comprehensive assessment of the injection effects.

More studies are needed to assess the efficacy of PS on various water stressors as well as the health and performance of diverse fish species. While this study focused on a single concentration, future research should include a range of doses to determine the dose‒response relationship. These findings provide deeper insights into the efficacy and optimal dosage of PS extract for mitigating thermal stress.

The effects of prolonged exposure to PS extract were not explored in this study because of the scope and timeframe of our experiments. However, we recognize the scientific interest in determining whether extended supplementation maintains its efficacy or introduces potential adverse effects. We propose that future studies are needed to investigate this aspect.

## Supplementary Information

Below is the link to the electronic supplementary material.

Supplementary Material 1

## Data Availability

The raw data that support the findings of this study are available from the corresponding author upon reasonable request.
