# Supplementary material for: Dietary Pelargonium Sidoides extract mitigates thermal stress in Oreochromis niloticus: physiological and immunological insights
Source: Vet Res Commun. 2025 Mar 24;49(3):152. doi: 10.1007/s11259-025-10705-z (PMC11933177; doi:10.1007/s11259-025-10705-z)
Supplement: Supplementary file 1 — Supplementary Material 1 [file 11259_2025_10705_MOESM1_ESM.docx]

**Table 1S. Details on the kits used and the serum biochemical, immunological, and antioxidant markers examined**

| **Cat No.** | **Method** | **Kits used** | **Parameters** |
| --- | --- | --- | --- |
| SP1001291 | Biuret. Colorimetric  wavelength 546 | Spinreact, Spain | Total protein |
| SP1001020 | Bromocresol green Colorimetric  wavelength 620 nm | Spinreact, Spain | Albumin |
| SP41274 | NADH. Kinetic UV. IFCC rec.  wavelength 340 nm | Spinreact, Spain | ALT |
| MD41264 | NADH. Kinetic UV. IFCC rec.  Wavelength 340 nm | Spinreact, Spain | AST |
| TK41214 | Pyruvate. Kinetic UV. DGKC.  Wavelength 340 nm | Spinreact, Spain | LDH |
| SP41041 | Urease - GLDH. Kinetic  Wavelength 340 nm | Spinreact, Spain | Urea |
| SP1001111 | Jaffé Colorimetric-Kinetic  Wavelength 505 | Spinreact, Spain | Creatinine |
| MBS704055 | Quantitative Competitive  Wavelength 450 nm  Sensitivity <1.56 ng/ml | MyBioSource Inc., USA | Cortisol |
| SP41011 | enzymatic colorimetric GOD-POD (Glucose Oxidase-Peroxidase)  Wavelength 505 nm | Spinreact, Spain | Glucose |
| TK41031 | enzymatic colorimetric GPO-POD (Glycerol Phosphate Oxidase-Peroxidase)  Wavelength 505 nm | Spinreact, Spain | TG |
| TK41021 | enzymatic colorimetric CHOD-POD  Wavelength 505 nm | Spinreact, Spain | CHO |
| TK1001096 | Direct. Enzymatic colorimetric  Wavelength 578 nm | Spinreact, Spain | HDL |
| MI41023 | Enzymatic colorimetric  Wavelength 600 nm | Spinreact, Spain | LDL |
| CSB-E15930Fh | Quantitative Competitive  Wavelength 450 nm  Sensitivity 187.5 μU/mL | Cusabio Biotech Co., Ltd.; China | GPx |
| CSB-E15929Fh | Quantitative Competitive  Wavelength 450 nm  Sensitivity 0.625 ng/mL | Cusabio Biotech Co., Ltd.; China | SOD |
| CSB-E15928Fh | Quantitative Competitive at  Wavelength 450 nm  Sensitivity 62.5 mU/mL | Cusabio Biotech Co., Ltd.; China | CAT |
| E-BC-K025-S | Colorimetric method  Detection Wavelength: 532 nm  Sensitivity: 0.38 nmol/mL | Elabscience Biotechnology, Inc. USA | MDA |

**Table 2S. Determination of total phenolic, total flavonoids content, and antioxidant activity of *Pelargonium sidoidis* roots (Ethanolic extract)**

| *Pelargonium sidoidis* roots (Ethanolic extract) | Total phenolic content (mg GAE/ g dry extract) | Total flavonoids content (mg QUE/ g dry extract) | Antioxidant activity by DPPH (µg TE/ml) |
| --- | --- | --- | --- |
|  | 27.7±0.03 | 66.1±0.18 | 0.971± 0.000  DPPH scavenging %: 39.7  IC_50_: 5.5 µg/ml |

Values are means ± SE, GAE; Gallic acid, QUE: Quercetin, DPPH: 1, 1- diphenyl-2-picryl hydrazyl free radical, TE: Trolox, which is a water-soluble analog of vitamin E. The Trolox Equivalent Antioxidant Capacity (TEAC) assay measures the antioxidant strength of a given substance by comparing it to Trolox, IC_50_: the concentration of sample required to inhibit 50% of the DPPH free radical

**
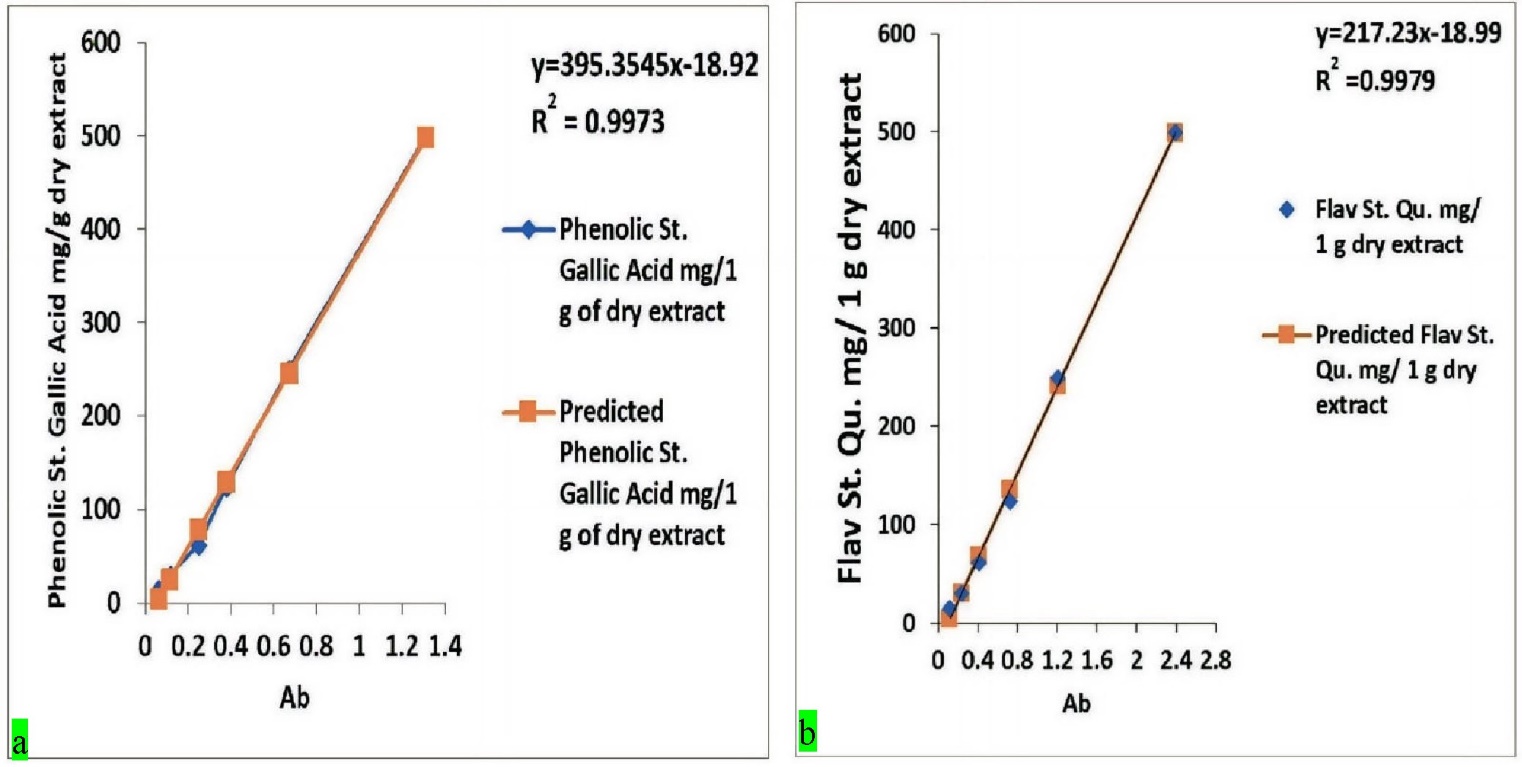
**

Fig. 1S The relationship between the absorbance (Ab) and the concentration of. a) total phenolic and b) total flavonoids contents in the ethanolic extract of PS-treated roots in mg per gram of dry extract

1. **Phenolic St. Gallic Acid Calibration Curve**

**X-axis:** Absorbance

**Y-axis:** Phenolic St. Gallic Acid (mg/g dry extract)

**Data Points:** Blue diamonds indicate the measured concentrations, while orange squares represent the predicted values based on the linear equation.

**Linear Equation:** y=395.35x−18.92

**R² Value:** 0.9973

**Explanation:**

The high R² value (0.9973) suggests a very strong linear relationship between the absorbance and the concentration of Phenolic St. Gallic Acid, meaning that the model accurately predicts the concentration based on the absorbance values.

The proximity of the blue diamonds to the orange squares indicates the precision of the model, with actual data points closely matching the predicted values.

1. **Flav St. Qu. Calibration Curve**

**X-axis:** Absorbance

**Y-axis:** Flav St. Qu. (mg/g dry extract)

**Data Points:** Blue diamonds indicate the measured concentrations, while orange squares represent the predicted values based on the linear equation.

**Linear Equation:** y=217.23x−18.99

**R² Value:** 0.9979

**Explanation:**

Similarly, the high R² value (0.9979) demonstrates a strong linear correlation between absorbance and the concentration of Flav St. Qu., affirming the reliability of the calibration model.

The closeness of the blue diamonds to the orange squares indicates the model's accuracy in predicting the actual concentrations based on absorbance.

**Table 3S Estimation of** **the antioxidant activity and IC_50_** **of the PS roots (ethanol extract)**

| Concentration (µl/ml) | *Pelargonium sidoides* root extract | | Ascorbic Acid Standard | |
| --- | --- | --- | --- | --- |
|  | OD Mean | DPPH scavenging % | OD Mean | DPPH scavenging % |
| 1000 | 0.082±0.004 | 94.9±0.002 | 0.032±0.001 | 98.0±0.000 |
| 500 | 0.113±0.004 | 93.0±0.002 | 0.062±0.003 | 96.1±0.002 |
| 250 | 0.199±0.002 | 87.6±0.001 | 0.096±0.002 | 94.1±0.001 |
| 125 | 0.318±0.001 | 80.3±0.001 | 0.140±0.001 | 91.3±0.000 |
| 62.5 | 0.419±0.002 | 74.0±0.001 | 0.241±0.003 | 85.0±0.001 |
| 31.25 | 0.526±0.002 | 67.3±0.001 | 0.390±0.001 | 75.8±0.001 |
| 15.625 | 0.657±0.001 | 59.2±0.001 | 0.509±0.002 | 68.4±0.001 |
| 7.8125 | 0.756±0.003 | 53.1±0.001 | 0.626±0.001 | 61.1±0.001 |
| 3.9 | 0.874±0.002 | 45.7±0.001 | 0.726±0.003 | 55.0±0.001 |
| 1.95 | 0.971±0.001 | 39.7±0.000 | 0.876±0.003 | 45.6±0.001 |
| 0 | 1.611±0.001 | 0.0±0.001 | 1.611±0.001 | 0.0±0.001 |
| IC50 (µl/ml) | - | 5.5 | - | 2.02 |

The values are means ± SE (n=3), DPPH: 1,1-diphenyl-2-picryl hydrazyl free radical, TE: Trolox equivalent (water-soluble vitamin E analog, and its antioxidant activity is used as a standard reference), IC_50_: the sample concentration needed to inhibit 50% of the DPPH free radical

**
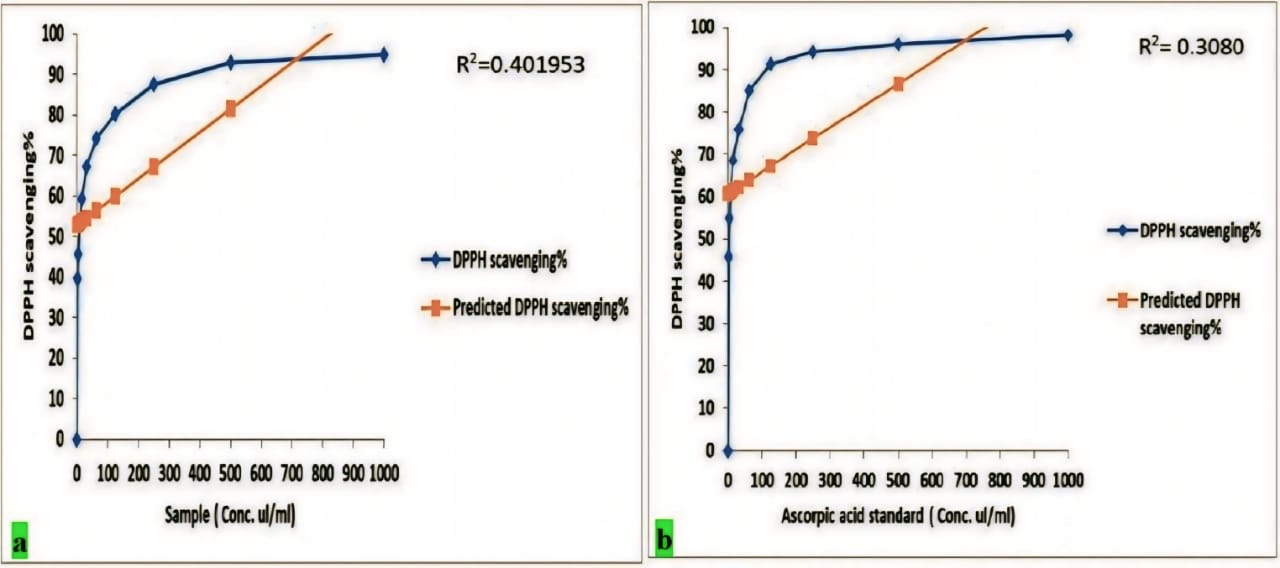
**

Fig. 2S Calibration Curve for antioxidant activity of PS roots (ethanol extract) determined via the DPPH radical scavenging method. DPPH: 1,1-diphenyl-2-picryl hydrazyl free radical, TE: Trolox equivalent (water-soluble vitamin E analog, and its antioxidant activity is used as a standard reference), IC_50_: and the sample concentration needed to inhibit 50% of the DPPH free radical

**Figure Explanation:**

The figure contains two calibration curves that illustrate the relationship between the concentration of samples and the DPPH scavenging percentage, which measures the total antioxidant capacity.

**a) Sample Calibration Curve**

**X-axis:** Sample concentration (µL/mL)

**Y-axis:** DPPH scavenging percentage (%)

**Data Series:**

Blue diamonds represent the actual measured DPPH scavenging percentages.

Orange squares represent the predicted DPPH scavenging percentages based on the linear model.

**R² Value:** 0.401953

**Description:**

The R² value of 0.401953 indicates a moderate correlation between the sample concentration and the DPPH scavenging percentage.

The blue diamonds depict the actual data points measured from the samples, while the orange squares represent the predicted values derived from the linear model.

The scatter of data points around the predicted line suggests variability in the antioxidant capacity measurements of the samples, highlighting areas where the model's prediction accuracy may vary.

**Ascorbic Acid Standard Calibration Curve**

**X-axis:** Ascorbic acid standard concentration (µL/mL)

**Y-axis:** DPPH scavenging percentage (%)

**Data Series:**

Blue diamonds represent the actual measured DPPH scavenging percentages.

Orange squares represent the predicted DPPH scavenging percentages based on the linear model.

- **R² Value:** 0.3080

**Description:**

The R² value of 0.3080 indicates a lower correlation between the ascorbic acid standard concentration and the DPPH scavenging percentage.

Similar to the left graph, the blue diamonds show the actual measured data points, while the orange squares represent the predicted values from the linear model.

The spread of data points around the predicted line highlights the variability in the antioxidant capacity measurements for the ascorbic acid standard, suggesting potential improvements in the model's accuracy.
